# Flight style and metabolism shape the tempo of genome evolution in birds

**DOI:** 10.1371/journal.pbio.3003884

**Published:** 2026-07-14

**Authors:** Yanzhu Ji, Lei Wu, Dongming Li, Shaohong Feng, Qi Fang, Ying Xiong, Yongbin Chang, Jacob C. Cooper, Xin Yu, Kai Zhang, Shiyu Tang, Huishang She, Huan Wang, Dezhi Zhang, Gang Song, Ping Fan, Jiaogen Zhou, Liang Ma, Yanhua Qu, Chenxi Jia, Catherine Sheard, James Andrew DeWoody, Joseph A. Tobias, Guojie Zhang, Weiwei Zhai, Fumin Lei

**Affiliations:** 1 State Key Laboratory of Animal Biodiversity Conservation and Integrated Pest Management, Institute of Zoology, Chinese Academy of Sciences, Beijing, China; 2 University of the Chinese Academy of Sciences, Beijing, China; 3 Hebei Key Laboratory of Animal Physiology, Biochemistry and Molecular Biology, Hebei Normal University, Shijiazhuang, China; 4 Evolutionary and Organismal Biology Research Center, Zhejiang University School of Medicine, Hangzhou, China; 5 Liangzhu Laboratory, Zhejiang University School of Medicine, Hangzhou, China; 6 Department of General Surgery of Sir Run Run Shaw Hospital, Zhejiang University School of Medicine, Hangzhou, China; 7 BGI-Shenzhen, Shenzhen, China; 8 Villum Centre for Biodiversity Genomics, Section for Ecology and Evolution, Department of Biology, University of Copenhagen, Copenhagen, Denmark; 9 College of Life Sciences, Sichuan Agricultural University, Yaan, China; 10 Henan Engineering Research Center of Bird Collisions, Zhengzhou Normal University, Zhengzhou, China; 11 Department of Biology, University of Nebraska at Kearney, Kearney, Nebraska, United States of America; 12 Negaunee Integrative Research Center, Field Museum of Natural History, Chicago, Illinois, United States of America; 13 College of Basic Medicine, Shaanxi University of Chinese Medicine, Xianyang, China; 14 School of Geography and Planning, Huaiyin Normal University, Huai’an City, China; 15 School of Biological Sciences, University of Aberdeen, Aberdeen, United Kingdom; 16 Departments of Forestry & Natural Resources and Biological Sciences, Purdue University, West Lafayette, Indiana, United States of America; 17 Department of Life Sciences, Imperial College London, London, United Kingdom; 18 Women’s Hospital, Zhejiang University School of Medicine, Hangzhou, China; University of Bath, UNITED KINGDOM OF GREAT BRITAIN AND NORTHERN IRELAND

## Abstract

As a hallmark of avian ecological innovation, powered flight has fundamentally shaped diverse aspects of birds. The energy demand of flight may have mutagenic impacts on genomes, influencing how fast genomes evolve. However, the relationship between flight, metabolism, and evolutionary rates remains relatively underexplored. Leveraging 363 newly available avian genomes from >90% of avian families, we quantified three distinct types of genomic evolutionary rates to capture a broad spectrum of mutational processes. By combining four flight-related traits and three metabolic metrics, we uncovered significant associations between flight style, metabolism, and multiple evolutionary rates. Next, using a causal inference framework, we demonstrated that metabolism accounted for 43.3% of the total effect between flight and evolutionary rate, underscoring its key role. Together, our findings establish a robust connection between flight, metabolism, and evolutionary rates, offering new insights into how key innovations and associated phenotypes shape the tempo of genome evolution.

## Abstract 2

Powered flight has fundamentally shaped diverse aspects of birds. This study reveals a robust connection between flight, metabolism, and evolutionary rates, suggesting that this influence extends to the tempo of genome evolution.

## Introduction

How and why molecular evolutionary rates (hereafter, “evolutionary rates”) vary among species has long intrigued biologists [1–3]. As one of the most commonly used evolutionary rates, nucleotide substitution rate is known to be related to a range of traits including body mass, generation time, and effective population size (*N*_e_; [4–6]). Different hypotheses have been proposed to explain these observations. For instance, animals with shorter generation times undergo more rounds of germline DNA replication and thus will accumulate more mutations [7,8]. Alternatively, it has also been hypothesized that basal metabolic rate, which is strongly related to body mass, is one of the factors that can contribute to the variation in substitution rate [9]. Mechanistically, Reactive Oxygen Species (ROS) derived from metabolic activities [10] can often induce single-nucleotide variations and insertion/deletions through defective DNA repair of different types of DNA lesions [11–14]. Additionally, effective population size could also influence mutation rates or the closely related substitution rates, as proposed by the “drift-barrier hypothesis” [15–17].

As one of the vertebrate lineages that evolved powered flight, birds exhibit diverse flight-related traits along with a wide range of evolutionary rates [6,18]. For instance, the lowest evolutionary rates have been observed in penguins (which are flightless) and in Procellariiformes (represented by albatrosses that utilize energy-efficient soaring or gliding flight; [6]). In contrast, birds that flap wings intermittently often fly with a wave-like or undulating flight track (e.g., woodpeckers and passerines) and have relatively high evolutionary rates [6]. It has also been observed that flapping or soaring flight styles have a great impact on metabolism in a few species [19–21]. In addition to the major distinction between soaring and flapping flight, the metabolic demand might be distinct for more nuanced differences in avian flight. For example, birds that exhibit undulating flight (i.e., flapping flight intermittent with non-flapping flight with folded wings) may have different energy demand compared to those with continuous flapping flight [21,22]. Birds from Galliformes and Tinamiformes have relatively small hearts, leading to limited aerobic performance (quick exhaustion, etc.) which induces ROS production [23,24]. These correlated observations lead to a compelling but untested hypothesis that different flight styles may impose different levels of metabolic demand and subsequently evolutionary rates in birds. However, the relationship between flight characteristics, metabolism, and evolutionary rates together with previously characterized life history traits has rarely been explored in a systematic manner.

In addition to the potential connection among flight characteristics, metabolism, and evolutionary rates, recent progress in studying mutational mechanisms provides an unprecedent opportunity to tackle this problem. For instance, deletions and single-nucleotide substitutions can arise from both DNA replication errors and metabolism-induced DNA damage [11,13,25]. This contrasts with the expansion and contraction of microsatellites, which are primarily attributed to replication slippage due to strand mispairing that are mechanistically different from other types of mutations [26,27]. Given that flight styles vary widely across different bird species—potentially impacting metabolism—an intriguing question emerges: how might differences in flight behavior, along with associated metabolic traits, contribute to variation in evolutionary rates?

**Fig 1:**
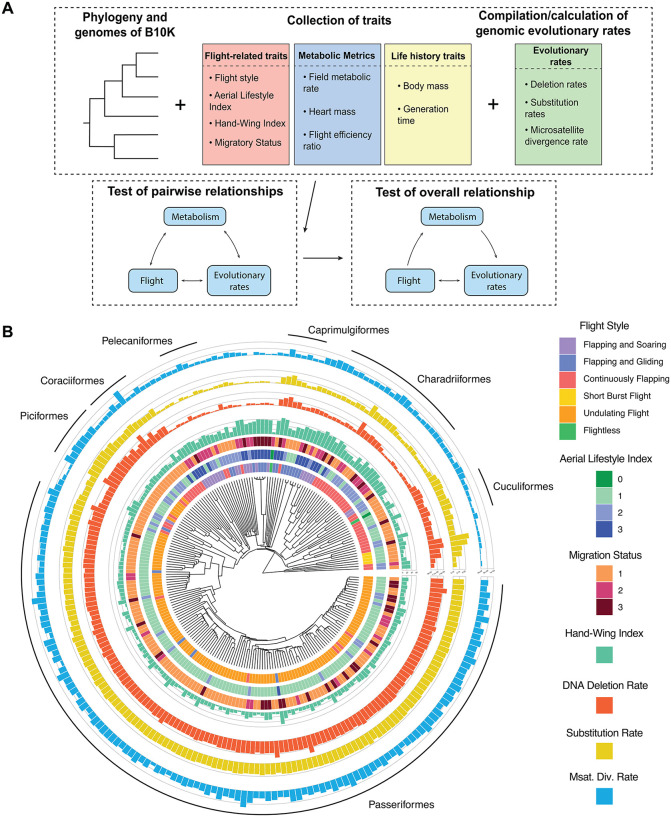
Study design and sampling. **(A)** Flowchart showing connection between genomic sample, traits compiled and relationships tested in this study. **(B)** Overview of flight characters and genomic evolutionary rates for 228 species sampled from 19 orders and 182 families of birds (note that only species with available data are shown). Colored bars at tip show (from inner to outer circles) flight style, Aerial Lifestyle Index (ALI), migration status, Hand-Wing Index (HWI), DNA deletion rate (per site per million years, or Mya), substitution rate (percent per Mya), and microsatellite divergence rate (Msat. Div. Rate.; repeat units per Mya). ALI describes the extent to which species rely on flight during foraging and daily activities from flightless species (0) to largely aerial species (3). Migration Status varies from sedentary species (1) to long-distance migrants (3). Species with missing data are excluded. Named orders contain >7 species. The data and code required to generate this Figure can be found in https://doi.org/10.5281/zenodo.20093624.

Here, we leverage genomes sequenced from 363 species sampled across 92% of avian families by the flagship Bird 10K (B10K) project [28,29]. Based on these genomes, we compile three types of evolutionary rates—substitution rates, deletion rates, and microsatellite divergence rates—to reflect different evolutionary processes or mutational mechanisms. We also compile four different types of flight characters for the same set of species, along with three metrics of metabolism. These datasets reveal that evolutionary rates are associated with flight characters, life history traits, and metabolic processes across a diverse sample of species, shedding light on the joint effects of flight and metabolism as factors shaping variation in evolutionary rates (Fig 1A).

## Materials and methods

### Calculation of genomic evolutionary distances and rates

To characterize the landscape of genomic evolutionary rates, we leveraged the rich genomic resources generated by the flagship Bird 10K Genomes Project (B10K). As the largest collaborative genome sequencing consortium in birds, the B10K project has released 363 avian genomes covering 92.4% families of birds [28,29]. To estimate a set of evolutionary rates with different mutational mechanisms, we selected three metrics of evolutionary rates. The first two rates, substitution rate and DNA deletion rate, reflected rates of evolutionary change in point mutations and DNA deletions, respectively [6,18]. The third evolutionary rate, microsatellite divergence rate, measures the change of orthologous microsatellite repeat units [18].

To calculate deletion rates, substitution rates, and microsatellite divergence rates, we relied on root-to-tip genomic evolutionary distances derived from previous studies [6,18]. Specifically, for each of these rates, we relied on measurements of deletion length, Jukes–Cantor distances (which were adjusted based on P distance), and microsatellite changes, respectively. Evolutionary rates were subsequently calculated by dividing the evolutionary distances by divergence time.

Both deletion length and P distances were calculated from the whole-genome alignment (HAL file) that was built by B10K project [30], with the distinction that deletion length was previously calculated by the authors [18], whereas P distance was inherited from a recent study [6]. Not only did the HAL alignment provide an excellent comparative genomics resource for all major avian lineages, it also included a “root” sequence, which referred to the ancestral sequence of all bird genomes included in the alignment and was originally built by aligning avian genomes to reptilian genomes to polarize avian genomic variations. This root sequence allowed direct comparison between any bird genome included in the HAL file and the ancestor of birds, thereby facilitating the calculation of deletion length and P distances.

To briefly recapitulate the technical details, deletion length was calculated by extracting multiple 1-kb blocks from the HAL file using the root sequence as the reference (S1 Fig; https://github.com/Secretloong/Cactus_Alignments_Tools). These blocks were randomly chosen from non-repetitive and non-genic regions based on genome annotations of *Gallus gallus*. After excluding sequences that were less than 500 bp of each block (representing poorly aligned sequences), deletion length per 1-kb block were calculated by summing the length of all gaps for each species. For each species, deletion length was then averaged across multiple blocks.

Due to computational challenges in deriving the deletion length from the whole genome alignments, we randomly sub-selected small portions (1-kb blocks) from the whole genome alignment without replacement for 100,000 times and computed the deletion length based on the subset of the genome. Deletion length estimates remain stable as the number of sampled blocks increased, supporting the sufficiency of 100,000 blocks (S1 Text).

The P distance we used in this work was also derived from the B10K alignment. Similar to our calculation of deletion length, P distance was calculated by constructing a gap-free whole-genome alignment from HAL and subsequently comparing each extant species to the ancestral (“root”) sequence [6]. Among the three metrics reported by Cole and colleagues [6], we chose P distance (supplementary Data 3, tab “Distances to MRCA 1”, column “P distance” in Cole and colleagues [6]) because it directly measures sequence divergence between each genome and the ancestor, consistent with the approach used for deletion length. We then converted the P distance into a Jukes–Cantor distance to correct for multiple substitutions.

Next, to reflect evolutionary distance of microsatellite change, we relied on our previous calculation of microsatellite length for each orthologous locus in each species. We further calculated microsatellite divergence length as the difference between the length of each locus compared to the length of ancestral locus, which was extracted from the avian whole-genome alignment (HAL file) as previously described. For each species, the microsatellite divergence rate was calculated by first dividing the divergence length of each locus by its ancestral length, then the species average was divided by the divergence time. Thus, microsatellite divergence rate reflects the rate of change of microsatellite length.

Finally, as all distances have been calculated relative to the ancestral (i.e., “root”) sequence, we calculated the evolutionary rates by dividing the distances by the root-to-tip branch length of the bird phylogeny (which represented the divergence time of the most recent common ancestor) that we used [28].

### Scoring and compiling of phenotypic traits

To combine genomic data with phenotypic data, we first compiled canonical metrics that describe life history traits or population-level parameters. We gathered body mass, generation time, and *N*_e_ from the existing literatures [31–33].

For flight-related traits, we specifically focused on those associated with energy metabolism. First, we relied on the state-of-the-art classifications of flight styles [21,22]. We also included a classification known as “short burst flight”, as recent studies suggested that birds with degenerated flight ability have switched their main energy source from lipids to carbohydrates and that they have relatively small hearts [34], with possible changes in oxidative stress and mutagenic consequences [35] and elevated metabolic rates [21]. Based on species accounts in *Birds of the World* [36], along with direct field experience in many cases, we classified the flight style of all study species into one of the following categories: “flapping and soaring”, “flapping and gliding”, “continuously flapping”, “short burst flight”, “undulating flight”, and “flightless”.

Operationally, flight style was classified as “flapping and soaring” if the species is known to use thermal updrafts (e.g., many raptors) or dynamic soaring in wind (e.g., albatrosses). “Flapping and gliding” birds refer to species that have prolonged non-flapping gliding phase (with wings stretched out) alternated by flapping phases (such as gulls and swifts). This differs from undulating flight (below), where the non-flapping phase involves folded or flexed wings rather than stretched wings. We used “continuously flapping” to indicate the type of flight that involves continuous flapping of wings without regular non-flapping phases. “Short burst flight” was used to categorize birds that routinely rest or forage on ground but occasionally would fly for a short period of time using flapping flight, such as pheasants and tinamous. Lastly, “undulating flight” represents an intermittent flight style usually exhibited by small passerines and woodpeckers, characterized by alternating flapping and non-flapping phases during which the wings are folded or partially flexed against the body, creating a wave-like trajectory (sometimes called bounding flight). Ultimately, the classification of flight styles reflects different adaptations of flight styles to different ecological niches. In order to keep scores consistent, we required two experts to score each species. The scores were further re-evaluated by another expert if the scores disagreed.

We additionally compiled three flight characters to complement flight style. We scored each bird to one of the four levels of aerial lifestyle index (ALI), with the scoring scheme detailed in Weeks and colleagues [37] (see S1 Text for details). Lastly, we incorporated published data on Hand-Wing Index (HWI) to represent wing shape, which is highly related to flight style [38,39] and compiled migratory status (i.e., resident, partial migrant or fully migrant) from AVONET to account for additional cost incurred by migratory species [31].

### Model building and model selection

Before constructing models to perform model selection, we first tested if any of the four flight characters are intercorrelated. We used phylogenetic Analysis of Variance (ANOVA) to detect the correlation between continuous variable (such as HWI) versus categorical variables (i.e., flight style, ALI, and migratory status), and used Fisher exact test for the correlation between categorical variables.

For each of the three evolutionary rates, we built an identical set of candidate models to evaluate whether adding flight traits improved model fit. To do that, we first constructed models that consisted of non-flight traits, i.e., models that included all possible combinations of one, two, or three variables of body mass, generation time, and *N*_e_ (7 models). We then added each of the four flight traits individually to each of the 7 base models, yielding 28 additional models (Table A in S1 Appendix). To alleviate possible confounding effects from the genome assembly, we also incorporated sequencing contig N50 values in each model.

Each model was fitted using phylogenetic generalized least squares (PGLS) with gls() function in the R package nlme, using the corPagel function to adjust each model for the amount of phylogenetic signal observed in residuals and specifying the method of “ML” (Pinheiro 2025). Models in each set were next selected based on AICc using AICcmodavg.

We considered models with ΔAIC < 2 as the best model(s). PGLS and phylogenetic ANCOVA (in which the flight styles were assigned as nominal character) were used to determine the correlation coefficients and level of significance of each best model. For best models with categorical predictors such as flight style and ALI, we used glht() function in the R package multcomp [40] to further make *post hoc* comparisons between each pair of the groups of the categorical variable (i.e., between “flapping and soaring” and “flapping and gliding”, “flapping and soaring” and “continuously flapping”, etc). The partial r^2^ of PGLS models were determined using rr2 package [41]. When a model with both life history traits and flight characters was identified as the best model, we used likelihood ratio tests to further compare the full model with the model with only life history traits using lrtest with the package lmtest [42].

### Sensitivity analysis

To evaluate if our results were influenced by various changes such as sampling of species or low-quality assemblies, we re-ran the model selection part (1) while excluding flightless birds, (2) while excluding species with lowest 10% contig N50 values. Our results could also be biased since the calculation of rates was dependent on the ancestral sequence reconstruction, which could be affected by rate heterogeneity. We cross-checked if model selection results were robust using substitution rates calculated from branch lengths of a phylogeny (i.e., without using the ancestral sequence; [6]).

To test if the model selection was affected by the ambiguity associated with flight style scoring in passerines or between certain categories, we also re-ran the model selection after we (1) assigned “continuously flapping” passerines to “undulating flight”, and (2) in all birds, combined “continuously flapping” and “undulating flight” birds to “flapping”, and combined “flapping and soaring” and “flapping and gliding” birds to “soaring and flapping”. We tested the robustness of our results by comparing the model selection results with those before these changes. We also compared the performance of two flight scoring systems based on the best models.

Additionally, as the diversification rate of species were reported to relate to the rates of morphological changes, it is possible that diversification rate is also related to genomic evolutionary rates. Therefore, we tested if the inclusion of diversification rate into one of the predictors would lead to consistent best models. We first calculated species-level diversification rates using the method in Jetz and colleagues [43], with the diversification rate of each species represented the median across the values calculated from 100 randomly sampled trees [43]. To incorporate the species-level diversification rate into our model, we followed an approach similar to the previous one (see section Model building and model selection), with diversification rates treated as one of the non-flight traits. We first built models excluding flight characters by creating the combinations among body mass, generation time, *N*_e_, and diversification rates, totaling 15 models. We next added each of the four flight characters to these models, resulting in 60 models. In cases where the gls() function resulted in unsuccessful convergence, we used phylolm to fit the PGLS models using the option ‘model = “lambda”’ with the default mode of “ML”.

### Correlation between energy metabolism, flight characters, and genomic evolutionary rates

To consolidate the relationship between flight characters and genomic evolutionary rates, we compiled metabolic metrics that directly quantify different aspects of metabolism. In particular, we collected (1) Field Metabolic Rate (FMR) that represented the total energy expenditure [44,45], (2) heart mass, which measures aerobic power and is related the ability of sustained flight [23], (3) the ratio between maximum aerobic mechanical power output continuously available given circulatory variables and the minimum power required to fly given the flight apparatus (*P*_ac_/*P*_min_, [46]). This metric examined flight efficiency and is also related to energy metabolism (thereafter termed   Flight Efficiency Ratio or FER).

To determine the measurement of energy metabolism that correlated with flight characters the most, we related each of FMR, heart mass, and FER to all three flight characters using PGLS or phylogenetic ANCOVA while accounting for potential effects of body mass. Notably, while FMR and heart mass were compiled on the level of species, FER was only available on the family level. Therefore, we summarized flight characters on family level to match the data on FER. We also sampled one species from each family to represent the family-level tree. We identified the measurements that showed significant correlations with flight characters for subsequent analyses.

We aimed to identify the relationship between metabolic metrics versus evolutionary rates. As the residuals of metabolic metrics, after controlling for body mass, determined whether the level of energetic efficiency, we first took the residuals of metabolic metrics when regressed them against body mass and *N*_e_. PGLS models were built test whether the residuals of metabolic metrics are related to each of the three evolutionary rates.

### Building all-inclusive models to predict evolutionary rates

We used mediation analysis to further explore the overall relationship between evolutionary rates, flight characters, and metabolic metrics. Our aim was to identify if the relationship between flight character and evolutionary rates was mediated by metabolic metrics. Note that we only focused on variables that showed significant relationship with others, i.e., deletion rates in evolutionary rates, flight style in flight characters, heart mass and FER in metabolic metrics. We analyzed mediation effect assuming metabolic metrics mediate the effect between flight style and deletion rates. Note that for heart mass and FER, we used residuals of them after regressing against body mass. We used non-parametric bootstrap to calculate *P* values for the mediation effect [47], and used R2_lik function in the package rr2 to decompose the *r*^2^ value of each predictor [48].

## Results

### Compiling genome and flight phenome datasets for birds

To systematically explore the relationship between genomic evolutionary rates and flight phenome, we obtained a dataset with both genomic sequences and phenotypic traits utilizing the genomic resources generated by the B10K project, existing literature on avian traits, and our own scoring of flight style and ALI (S1 Data).

In the end, we categorized all 363 birds into “flapping and soaring” (*n* = 31), “flapping and gliding” (*n* = 30), “continuously flapping” (*n* = 96), “undulating flight” (*n* = 168), “short burst flight” (*n* = 24) and “flightless” (*n* = 14). Together with other flight traits, we found that these four flight traits are intercorrelated (S2 Fig). Nevertheless, they reflect complementary roles and dimensions of flight behavior, providing a general overview of the avian flight phenome (Fig 1).

### Flight style was among the best predictors that explain deletion rates and substitution rates

To test if any of the flight traits predict genomic evolutionary rates, we built three sets of models to “explain” each evolutionary rate, each of which was composed of several PGLS models. In each set of models, we included basic models with non-flight traits as well as advanced models that further included flight characters. As the flight characters were correlated, we included one flight trait at a time (Table A in S1 Appendix). By testing different models hierarchically, we examined whether the best model included flight characters.

**Fig 2:**
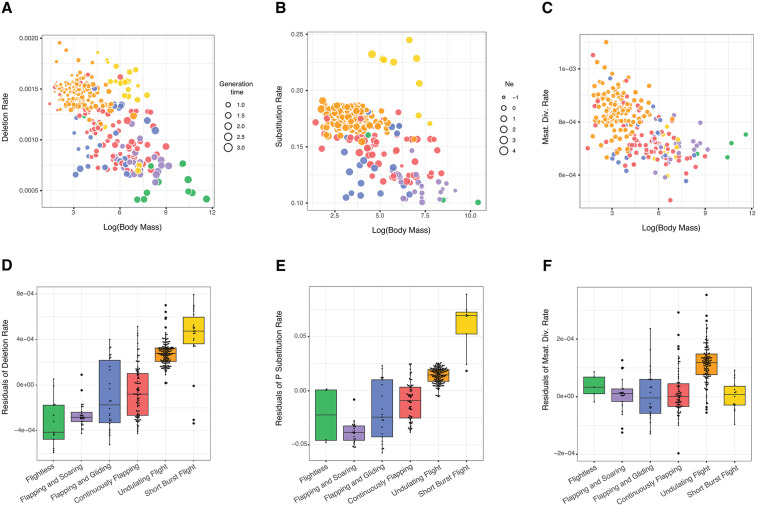
Relationship between genomic evolutionary rates and flight style. Scatterplots show the relationship between body mass and **(A)** deletion rate (323 sp.), **(B)** substitution rate (304 sp.), and **(C)** microsatellite divergence rates (272 sp.), with flight styles colored differently (see d-f for color codes). **(D–F)** shows boxplots of the residuals when regressing each of the genomic evolutionary rates with body mass, showing different trends among flight styles for (D) deletion rate, (E) substitution rate, and (F) microsatellite divergence rate. Note that flight styles were treated as nominal variables during phylogenetic ANOVA tests, but the results were ordered for better visualization. The data and code required to generate this Figure can be found in https://doi.org/10.5281/zenodo.20093624.

Our results showed that models that included flight style were consistently ranked as best models that explain deletion rates and substitution rates (ΔAICc < 2, Table B in S1 Appendix). For deletion rates, the best models included all three non-flight traits, with body mass and flight style contributing significantly to the best models (PGLS, *p* < 0.05). This was consistent with the partial *r*^2^ for each predictor in a PGLS model with all three non-flight traits and flight style, since flight style and body mass explained the largest part of variation for deletion rates (Table 1; S2H Fig). Additionally, flight contributed significantly to the model (phylogenetic analysis of covariance, or phylogenetic ANCOVA, *F* = 8.97, *p* < 0.001; Fig 2; Table C in S1 Appendix), and the inclusion of flight style significantly improved model fit (Likelihood Ratio Test, or LRT, *p* < 0.001). For substitution rates, the best model included body mass, *N*_e_, and flight style with all predictors significantly contributing to the best model (body mass: PGLS, coef. = −0.002, *p* < 0.001; *N*_e_: PGLS, coef. = −0.0006, *p* < 0.05; flight style, phylogenetic ANCOVA, *F* = 12, *p* < 0.001; Fig 2; Table D in S1 Appendix). The partition of *r*^2^ showed that flight style, *N*_e_ and body mass explained the variance of substitution rates more evenly when compared to the model of deletion rates (Table 1; S2H Fig). For microsatellite divergence rate, the model with body mass outperformed other models (Table B in S1 Appendix). Inclusion of flight style did not significantly improve the model fit when compared to a model with body mass as the predictor (LRT, *p* value > 0.05).

**Table 1:** Partial *R*^2^ values of predictors that explain deletion rates and substitution rates as determined by best models.

Full model: del. rate ~ body mass + gen. time + *N*_e_ + flight style	
Predictor	Partial *R*^2^
Body mass	0.033
Gen. time	0.014
*N*_e_	0.007
Flight style	0.116
Full model: substitution rate ~ body mass + *N*_e_ + flight style	
Predictor	Partial *R*^2^
Body mass	0.077
*N*_e_	0.098
Flight style	0.19

### The overall conclusion is robust against various changes in inputs

To examine the robustness of our findings, we further varied the models by (1) exclusion of flightless birds due to the inconsistent trend of HWI for flightless birds (see S1 Text for details), and (2) exclusion of relatively low-quality assemblies with low N50. Across both scenarios, we find that the model with flight traits consistently ranked as the top model that predicted deletion rates and substitution rates (Tables E and F in S1 Appendix). Notably, for the model selection of microsatellite divergence rate when excluding low-quality assemblies, we found models with ALI are among the best models (Table F in S1 Appendix). Nevertheless, ALI had insignificant contribution to microsatellite divergence rates (phylogenetic ANCOVA, *p* > 0.05). For substitution rates measured using branch lengths [6], flight style also contributed significantly (Table G in S1 Appendix). The close agreement between Cole’s substitution rates and the root-to-tip based rates suggested that the rate heterogeneity was qualitatively unlikely to bias the results. Together, these results highlighted the importance of flight traits, and especially flight style, in shaping deletion rates and substitution rates, but not microsatellite divergence rates.

Another uncertainty of our modeling came from the scoring of flight styles. Despite our rigorous efforts in categorizing the flight style, flight style occurs on a continuous spectrum in nature. This is particularly prominent in Passeriformes, where the distinction between “undulating flight” (157 out of 173 species) and “continuously flapping” (*n* = 8) is less well-defined. To explore the robustness of our conclusion, we re-assigned all “continuously flapping” passerines to “undulating flight” as the vast majority of passerines use “undulating flight”, and then re-performed the model selection. The results showed that contribution of flight style to evolutionary rates remained unchanged (Table H in S1 Appendix).

An alternative way to take into account uncertainties in flight style among passerines is to focus on migratory status, which is more easily scored. Moreover, passerine migration was reported to be related to synonymous substitution rate of mitochondrial DNA [49]. Interestingly, when testing if body mass and migration explained the genomic rates in Passerines, deletion rates were significantly different across migration categories (phylogenetic ANCOVA: *F* = 20.11, *p* < 0.001), whereas substitution rates showed less significant signals (substitution rates, *F* = 2.00, *p* = 0.13). When plotting deletion rates and substitution rates against the migration status (e.g., “1” for sedentary to “3” for fully migratory), we observed a general increase in these two evolutionary rates for fully migratory species (S3 Fig). Thus, the analyses on migration status in Passerines further supported our conclusion that species with more active migratory behavior have elevated evolutionary rates compared with resident species (i.e., migration status scored as “1”).

We also performed the model selection after merging flight styles into smaller number of categories to test the robustness of our results and the necessity to use finer-graded flight styles. After grouping “flapping and soaring” and “flapping and gliding” into “soaring and gliding”, and grouping “continuously flapping” and “undulating flight” into “flapping”, we found the same pattern still held, in that flight style was among the best models for deletion rates and substitution rates, but not for microsatellite divergence rates (Table I in S1 Appendix). Utilizing this combined 4-type flight style, we also tested which flight style categorization predict the rates best. When we compared the previous fine-graded flight style (6 types) with the combined 4-type flight style, we found that whereas the two flight style systems performed equally for deletion rates, the fine-graded flight style performed better than the 4-type flight style (Table J in S1 Appendix), indicating the necessity to classify flight styles into finer categories.

Finally, as diversification rates have been shown to be related to morphological traits [50,51], we investigated if the addition of diversification rates affected our modeling results. After expanding our 35-model set to a 75-model set after the inclusion of diversification rates (see Materials and methods), we found that diversification rate contributed significantly to deletion rates and substitution rates, but not microsatellite rates (Table K in S1 Appendix). The statistical significance of flight style to deletion rates, substitution rates, and microsatellite divergence rate remained unchanged (Table K in S1 Appendix).

### Multiple metabolic traits are related to flight style

We have so far revealed a robust connection between flight style and evolutionary rates, supporting our hypothesis that flight style may have an impact on evolutionary rates. However, it is yet unknown if this connection could be attributed to the metabolic difference among flight styles. Therefore, we compiled three metrics from the literature, i.e., FMR, heart mass and FER, that directly quantify different aspects of metabolism [23,44–46], and tested if any of these metrics were correlated with flight characters.

**Fig 3:**
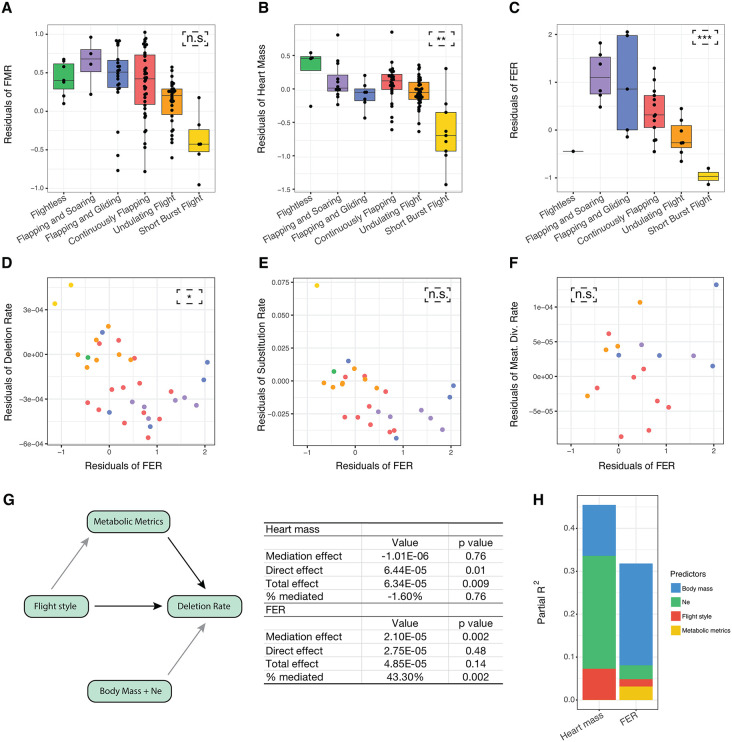
Relationships among flight traits, metabolism and evolutionary rates. Panels show **(A–C)** how flight style is related to three metabolic metrics, **(D–F)** how one of the metabolic metrics (Flight Efficiency Ratio, FER) is further related to genomic evolutionary rates, and **(G–H)** the relationship between metabolic metrics, flight style and deletion rates. Panels A–C show the relationship between flight style and residuals of (A) Field Metabolic Rate (FMR; 107 sp.), (B) heart mass (97 sp.), and (C) FER (33 families), with the significance of phylogenetic ANCOVA labeled in each boxplot. Panels D–F illustrate the scatterplots between residuals of evolutionary rates and residuals of FER after controlling for body mass and phylogeny (D: 33 families, E: 27 families, F: 17 families). Statistically significant relationships are represented by asterisks (* = *p* < 0.05); non-significant relationships are labeled “n.s.”. Note that residuals of evolutionary rates are used for visualization purposes (models were parameterized with rates not residuals). (G) Design and results of mediation analyses using either heart mass or FER to explain deletion rates. (H) Decomposition of *r*^2^ by body mass, flight style, and metabolic metrics. The data and code required to generate this Figure can be found in https://doi.org/10.5281/zenodo.20093624.

As flight style is the leading flight character that has been repeatedly identified to explain deletion rate and substitution rate, we then regressed each of the metabolic metrics against flight style and found that both heart mass and FER were significantly related to flight style (Table L in S1 Appendix; Fig 3A–3C). Together, these results showed that heart mass and FER were best related to flight style, highlighting the strong connection between energy metabolism (as represented by heart mass and FER) and flight style.

### Metabolic traits also predict deletion rates

Our previous analysis indicated strong links between flight style and evolutionary rates as well as between flight style and metabolic metrics, naturally implying a potential link between metabolism and evolutionary rates. To explore this relationship, we regressed each of the evolutionary rates against residuals of heart mass and FER (i.e., the residuals after regressing heart mass and FER against body mass). We found that deletion rates are significantly explained by the FER as well as heart mass (Table M in S1 Appendix; Figs 3D and S4A). These observations imply a strong connection among deletion rates, flight style and metabolic rates (e.g., FER and heart mass). In contrast, substitution rates are not explained by any of the metabolic rates, even though substitution rates are strongly linked to flight style (Figs 3E and S4B; Table M in S1 Appendix). These observations suggest that there are other factors contributing to substitution rates in addition to DNA replication errors and metabolism. Microsatellite divergence rates consistently show no link to flight style or metabolic traits (Figs 3F and S4C; Table M in S1 Appendix). Taken together, by establishing the relationship between metabolic traits and evolutionary rates, our results provided direct support for the link between energy metabolism and deletion rates.

### Metabolic traits mediate the link between flight style and deletion rates

So far, we have systematically explored the relationships between pairs of the variables among flight characters, metabolic metrics and evolutionary rates. How they could jointly act in a full model could provide a deeper insight into the causative relationship among all the variables. Particularly, we hypothesize that flight style may influence evolutionary rates via metabolic metrics. Therefore, using a causal inference framework known as mediation analysis [52], we first aimed to identify whether the relationship between flight style and deletion rates is mediated by any of the metabolic traits that were significantly related to deletion rates (i.e., heart mass and FER; Fig 3G). Using mediation analysis, we found that out of the total effect between flight style and deletion rates, FER significantly mediated 43.3% of the total effect (*p* < 0.05), whereas the mediation effect of heart mass was insignificant (Fig 3G). These results showed that the mediation effect of metabolic traits (i.e., heart mass and FER) can vary significantly. Additionally, the partial mediating effect suggests that additional factors could mediate the relationship between flight style and deletion rates.

The causal-inference analysis established the mediating role of each metabolic metric on the relationship between deletion rates and flight style. However, how different factors including body mass, flight style and metabolic metrics could jointly explain the deletion rates and the relative contributions of these factors remained unknown. To calibrate these contributions, we built multiple regression models with body mass, flight style, and one of the metabolic metrics (i.e., heart mass and FER) as predictors, and calculated the pseudo *R*^2^ for each explanatory variable. In these models, body mass and *N*_e_ consistently explained larger parts of the variation in deletion rates. When relating with heart mass and FER, body mass explained 11.9% and 23.7% of the variation, respectively, while *N*_e_ explained 26.3% and 3.2% (Fig 3H). For the multiple regression with heart mass, flight style explained almost all of the remaining variation (7.3%) while heart mass didn’t explain much (0.02%). In comparison, the decomposition of *r*^2^ with FER showed that FER explained a relatively larger proportion of variation (3.1% for FER and 1.7% for flight style; Fig 3H). For the first time, these results shed light on an interesting and tiered landscape wherein evolutionary rates are shaped by a suite of macro to micro-level determinants operating at varying hierarchical levels.

## Discussion

Taking advantage of the rich genomic resource from B10K project with more than 300 whole genomes from major families of birds, we first calculated multiple evolutionary rates including substitution rates, deletion rates, and microsatellite divergence rates, covering multiple mutational processes. We also curated several flight characters including flight style, HWI, ALI as well as migratory status, capturing complementary dimensions of avian flight phenotypes. Linking flight characters to evolutionary rates, we uncovered strong links between flight phenotypes and multiple evolutionary rates with the strongest association between flight style and substitution (or deletion) rates. Interestingly, microsatellite divergence rates are not related to flight traits. As powered flight has a profound impact on avian metabolism, we hypothesized that metabolic traits might also affect evolutionary rates. By extracting multiple metabolic traits such as heart mass and FER, we subsequently uncovered significant links between metabolic metrics and flight style, as well as between metabolic traits and evolutionary rates. In order to dissect the complex relationship between these factors, we employed a causal inference framework and found that a substantial proportion of flight-induced evolutionary rate variation is mediated through metabolic traits. Taken together, our study provides a general synthesis of the role of flight characters in evolution, emphasizing the inherent connection between macroevolutionary traits (e.g., flight style and FER) and evolutionary rates.

Our findings linking avian locomotion and molecular evolution rates shift our understanding of how avian flight affects molecular evolution compared with the conclusions of previous studies [3,53,54]. For example, among flighted birds, we found that species with less efficient flight (e.g., “short burst” and “undulating” flight) tend to have higher deletion rates and substitution rates than those with efficient flight (e.g., soaring or gliding birds). This pattern is supported by a recent study showing that passerines with shorter migratory distance (and thus, in general, less efficient flight ability) have higher mitochondrial synonymous substitution rates [49]. When calculating substitution rates in mtDNA genes using synonymous sites in our dataset, we detected a similar relationship (S1 Text in Supplementary Material). Unlike most previous studies, our dataset is specifically curated to reflect flight-related traits rather than broadly sampling traits that cover life history, morphology, ecology, and geographical features. Moreover, we use novel predictors—flight style—which may provide a more thorough assessment of flight-associated effects, combining variation in both wing shape (e.g., HWI) and wing loading [38].

Our study identifies a previously undescribed connection between flight traits and evolutionary rates, which aligns with the broader framework of the Metabolic Theory of Ecology which predicts that metabolic rates set the rates of ecological processes from life history traits (including substitution rates) to population-level interactions [9,55,56]. Moreover, our results that deletion rates and substitution rates are the highest in “short burst flight”, followed by “undulating flight”, “continuously flapping flight”, and so on, suggest that energy efficiency associated with flight styles may be the key determinant underlying different flight styles. Birds from Galliformes and Tinamiformes are reported to have relatively smaller hearts and thus have limited aerobic performance [23,24]. The associated quick exhaustion and higher post-exercise oxygen consumption could contribute to their higher evolutionary rates. Similarly, birds with undulating flight usually have round wings (S2B Fig), the wings that are inefficient for flight but improve maneuverability. Birds that mainly utilize “continuously flapping”, “flapping and soaring” or “flapping and gliding” have relatively larger hearts, and their wings are either slender or broad that improve energy efficiency by reducing drag or by being able to soar. Ultimately, different flight styles (and the associated morphologies) are closely linked to adaptations to different habitats [57], where the energy efficiency in turn influences the rates of genomic evolution.

In addition, our results on deletion rates provide a novel connection to genome size evolution. For example, the strong link between deletion rates and flight style suggests that certain phenotypic traits, including small body mass or energetically costly flight styles such as “short burst flight” and “undulating flight”, may lead to higher deletion rates, and thus smaller genomes. This prediction matches several observations. For example, hummingbirds, the lineage of birds with the smallest body mass and highly aerial lifestyles, have the smallest genomes among birds [58]. These observations also match previous studies that have linked small genome sizes in birds to high metabolic intensity, represented by flight muscle size and the ratio of heart mass to body mass [59].

It has long been hypothesized that avian genome size has evolved as an “adaptive trait” associated with flight and the higher metabolic demand of flying birds [60–62]. Under this hypothesis, birds with more frequent flapping flight may show smaller genome sizes, faster deletion rates, and smaller microsatellite divergence rates. However, our results contradict this prediction in that the “continuously flapping” birds have faster deletion rates, but not smaller microsatellite divergence rates. This discrepancy suggests factors other than genome size adaptation may underlie these patterns. Our results are compatible with a more general explanation that deletion rates and microsatellite divergence rate evolve differently because they are associated with distinct mutagenic mechanisms. For the generation of deletions, the additional involvement with metabolism could explain their correlation with flight style. Future studies should test whether flight or metabolic characters can induce a biased mutation (i.e., more deletion) or whether mutations coupled with subsequent adaptive evolution have driven the genome size evolution [63]. Taken together, flight characters and metabolic traits seem to influence both deletion rates and genome size evolution in birds.

Finally, our study sheds important light on different mechanisms underlying evolutionary rates. First, errors introduced by metabolic activity (e.g., ROS) may be a common driving mechanism for deletion rates, as reflected in the strong link between metabolic traits and deletion rates in our analysis. In contrast, microsatellite variations which were generated via DNA replication errors [64,65] are not related to flight characters or metabolic traits. This character of microsatellites also makes them reliable markers for calibrating cell division number [66]. Substitution rates may have been affected by both mechanisms mentioned above, consistent with recent observations that substitutions in germline cells included mutations that closely track cell division number as well as those reflecting DNA damage [67]. Thus, our study uncovers a highly structured, network-like relationship among evolutionary rates, life history traits, flight characters, and metabolic traits (S5 Fig), highlighting the complex impact of flight and metabolism on the tempo of genome evolution.

## Supporting information

S1 Fig Examples of alignment extracted from “HAL” file, showing how deletions are retained in the alignment, while insertions are omitted from the alignment. (TIF)

S2 Fig Intercorrelation among flight characters, and the contribution (partial *r*^2^ values) of deletion rates and substitution rates as explained by the predictors in the best models. **(A)** shows the overall relationship among Hand-Wing Index (HWI), flight style, Aerial Lifestyle Index (ALI), and migratory status, with the level of statistical significance (*** *p* < 0.001) indicated on each relationship. **(B–G)** show each of the pairwise comparisons, with labels corresponding to arrows in (A). The pairwise tests that are insignificant are labeled with “n.s.” in (B–D). **(H)** shows the contribution (partial *r*^2^ values) of deletion rates and substitution rates as explained by the predictors in the best models. The data and code required to generate this Figure can be found in https://doi.org/10.5281/zenodo.20093624.
(TIF)

S3 Fig In Passerines, the change of residuals of (A) deletion rates, (B) substitution rates and (C) microsatellite divergence rates (after correcting for body mass and contig N50) with different migratory status. The data and code required to generate this Figure can be found in https://doi.org/10.5281/zenodo.20093624.
(TIF)

S4 Fig Scatterplots between residuals of heart mass and residuals of (A) deletion rates, (B) substitution rates, and (C) microsatellite divergence rates. Statistically significant relationship is represented by stars (* indicates *p* < 0.05) whereas insignificant relationships are represented “n.s.”. Note that we used residuals of evolutionary rates only for visualization purposes, and the models were constructed with rates (instead of residuals). The data and code required to generate this Figure can be found in https://doi.org/10.5281/zenodo.20093624.
(TIF)

S5 Fig Summary of the relationships among flight traits, metabolic metrics, and evolutionary rates. This figure lists all factors considered in the paper, but some factors were chosen for further analyses (blue backgrounds). For example, for flight traits, we chose flight style among all four traits from model selection results to interrogate the relationship between flight style and metabolic traits. Statistically significant results are linked by solid lines. Methods that were used for the results are annotated adjacent to the lines.
(TIF)

S1 Appendix Tables A – M. (DOCX)

S1 Text Supplementary text covering deletion length calculation, scoring aerial lifestyle index, impact of flightless birds on modeling results, and model selection results using substitution rates calculated based on mitochondrial DNA. (DOCX)

S1 Data Data of flight style and ALI for the species included in this study. (XLSX)

## Abbreviations

ALI: aerial lifestyle index
ANOVA: Analysis of Variance
ANCOVA: Analysis of Covariance
FER: Flight Efficiency Ratio
FMR: Field Metabolic Rate
HWI: Hand-Wing Index
LRT: Likelihood Ratio Test
PGLS: phylogenetic generalized least squares
ROS: Reactive Oxygen Species
