# Supplementary figures and images for: Flight style and metabolism shape the tempo of genome evolution in birds
Source: PLoS Biol. 2026 Jul 14;24(7):e3003884. doi: 10.1371/journal.pbio.3003884 (PMC13367715; doi:10.1371/journal.pbio.3003884)

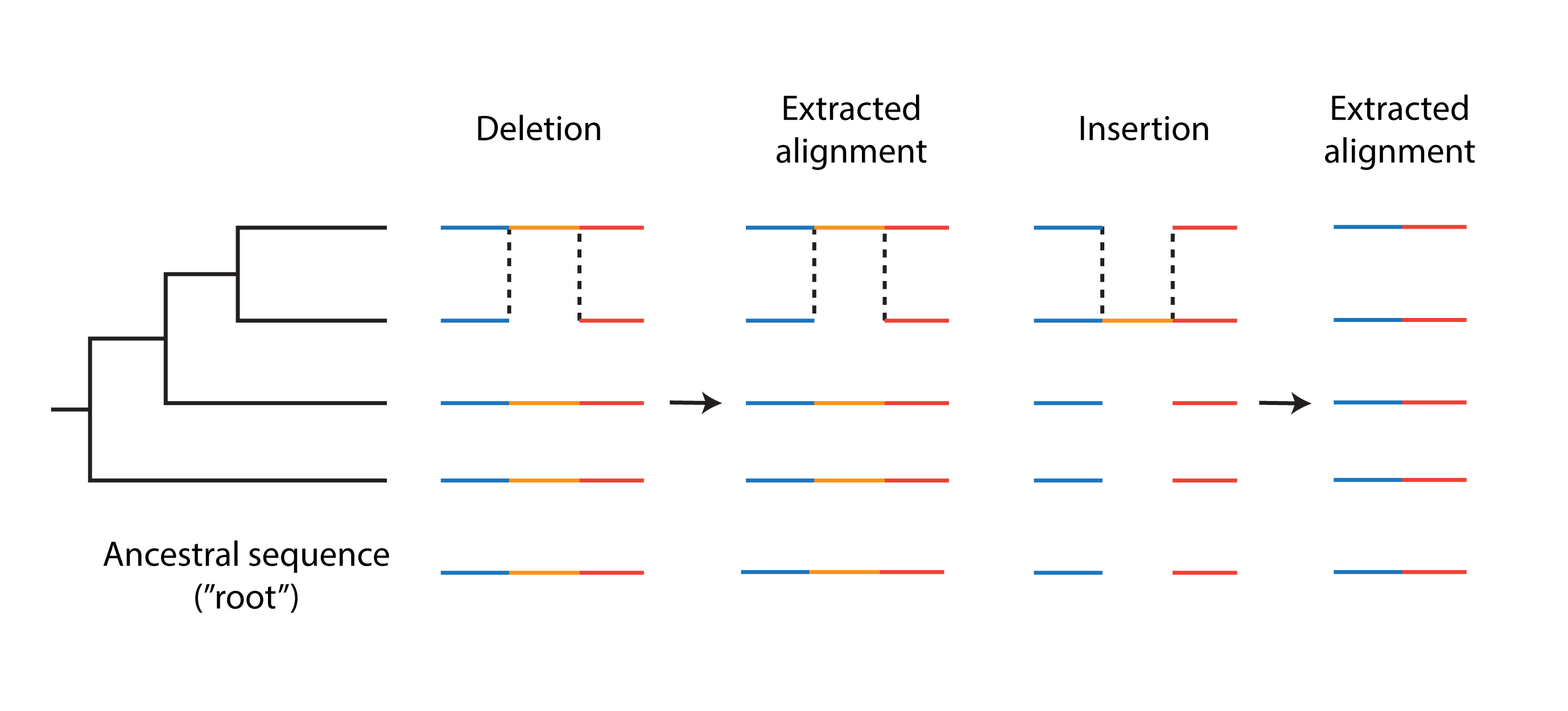

Supplement: S1 Fig — (TIF) [file pbio.3003884.s001.tif]

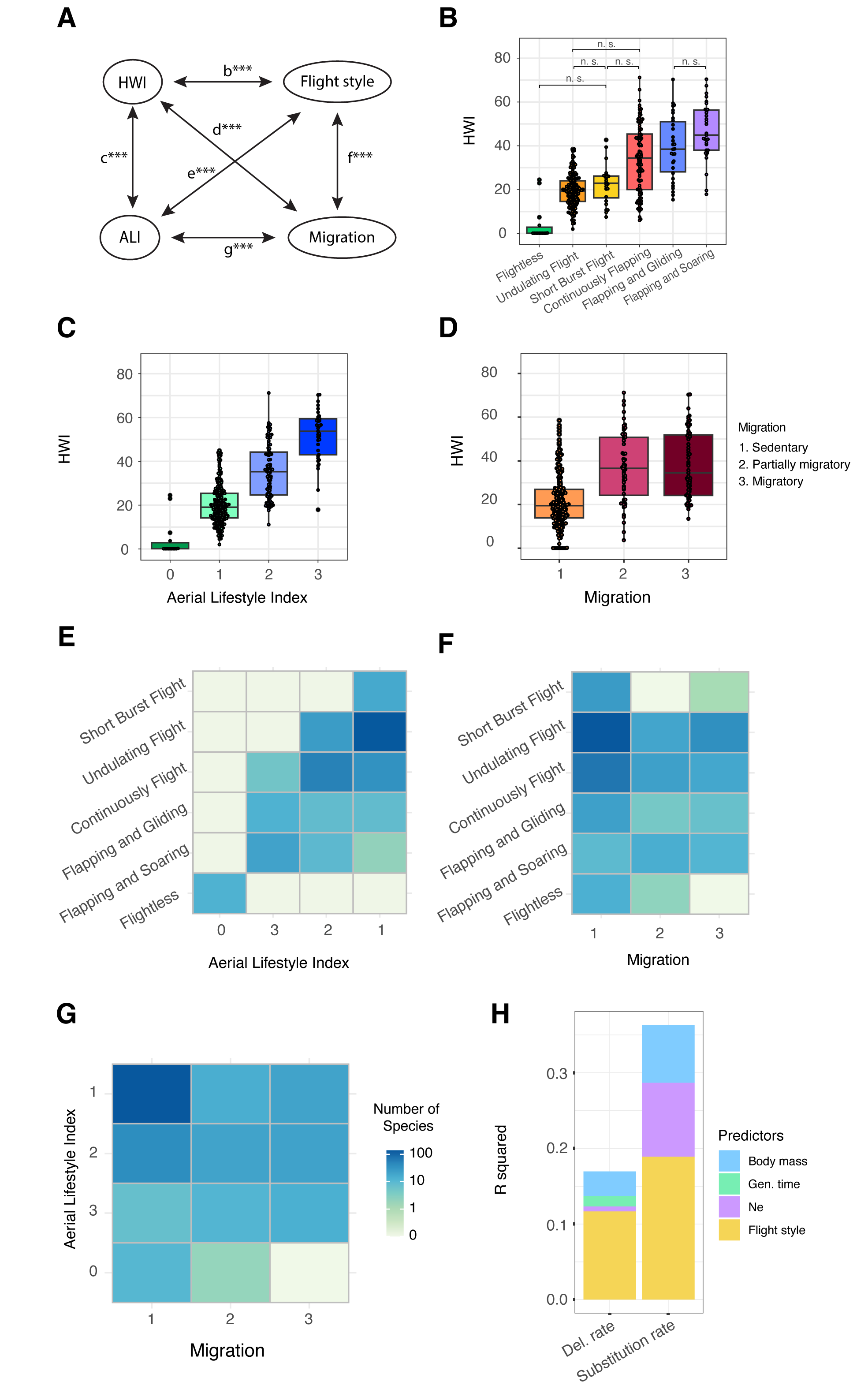

Supplement: S2 Fig — (A) shows the overall relationship among Hand-Wing Index (HWI), flight style, Aerial Lifestyle Index (ALI), and migratory status, with the level of statistical significance (*** p < 0.001) indicated on each relationship. (B–G) show each of the pairwise comparisons, with labels corresponding to arrows in (A). The pairwise tests that are insignificant are labeled with “n.s.” in (B–D). (H) shows the contribution (partial r2 values) of deletion rates and substitution rates as explained by the predictors in the best models. The data and code required to generate this Figure can be found in https://doi.org/10.5281/zenodo.20093624. (TIF) [file pbio.3003884.s002.tif]

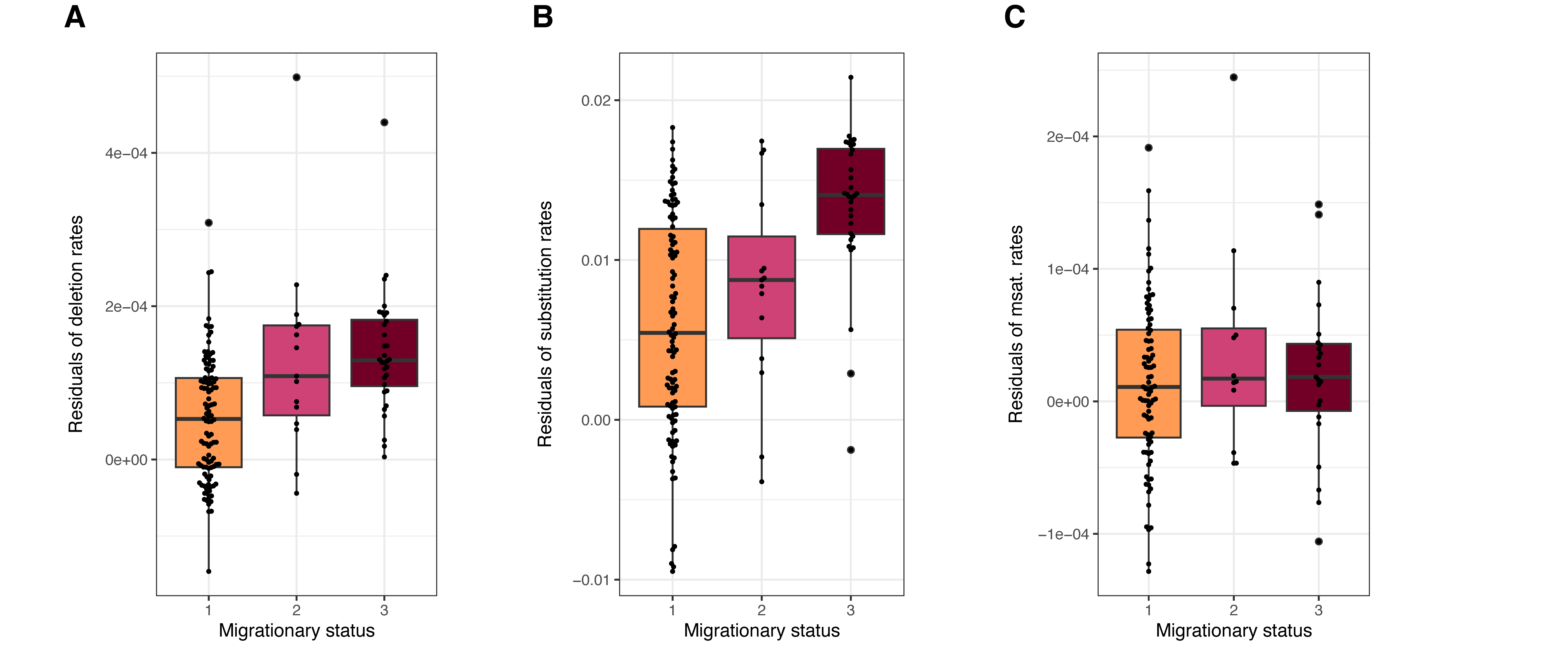

Supplement: S3 Fig — The data and code required to generate this Figure can be found in https://doi.org/10.5281/zenodo.20093624. (TIF) [file pbio.3003884.s003.tif]

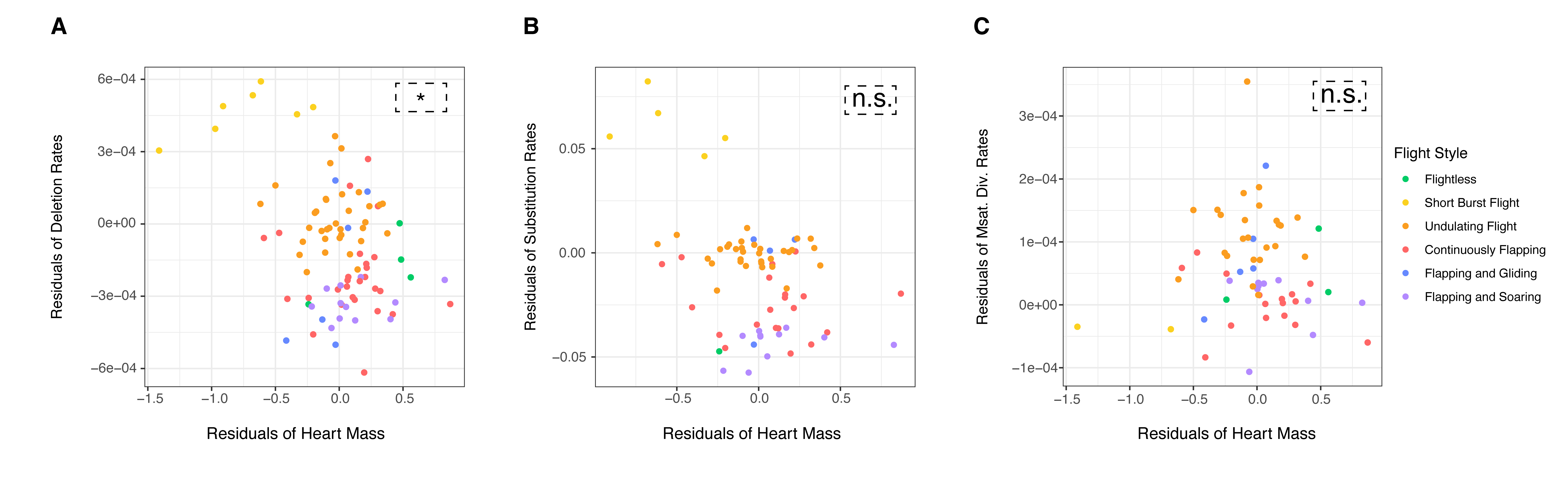

Supplement: S4 Fig — Statistically significant relationship is represented by stars (* indicates p < 0.05) whereas insignificant relationships are represented “n.s.”. Note that we used residuals of evolutionary rates only for visualization purposes, and the models were constructed with rates (instead of residuals). The data and code required to generate this Figure can be found in https://doi.org/10.5281/zenodo.20093624. (TIF) [file pbio.3003884.s004.tif]

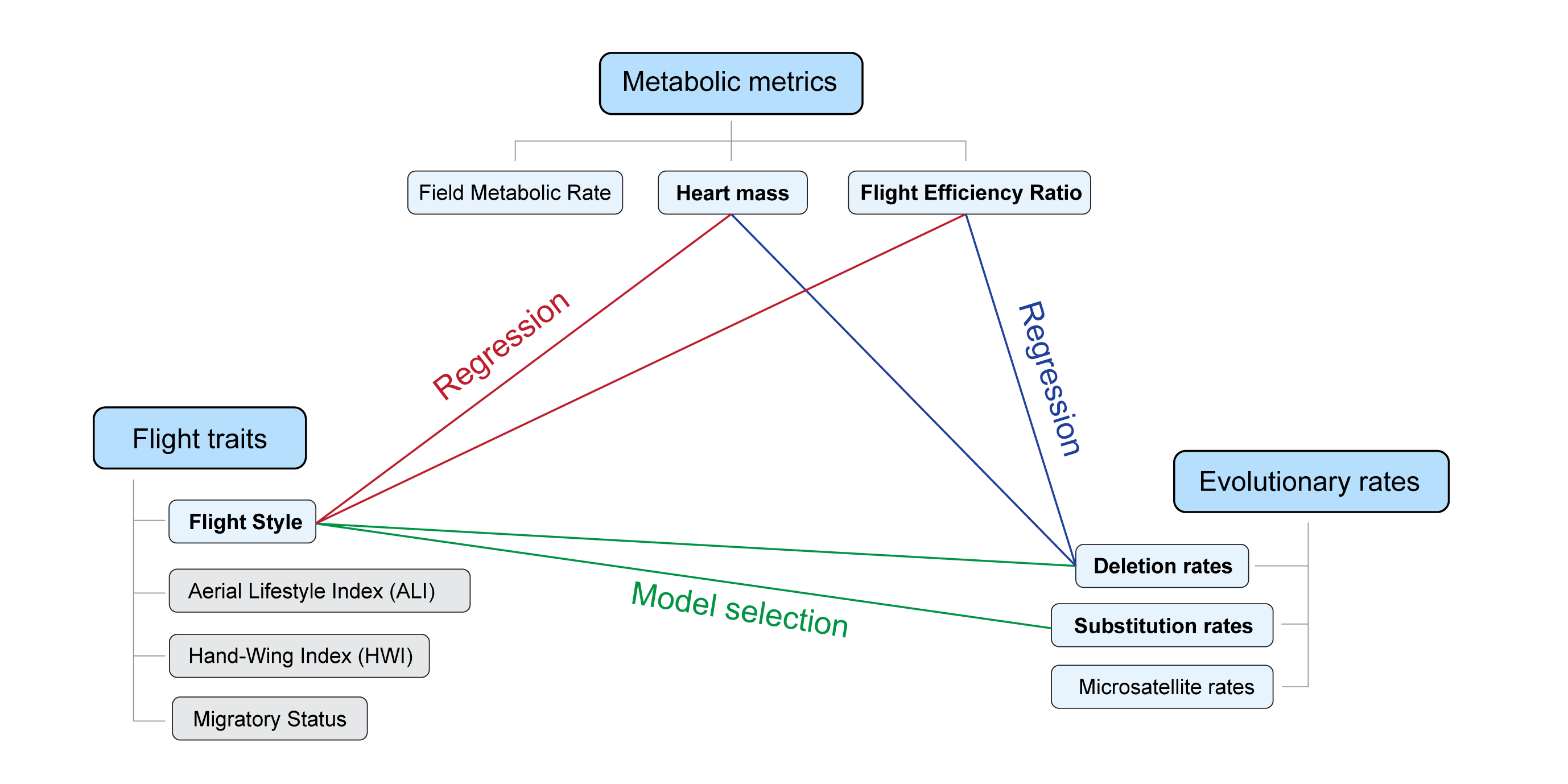

Supplement: S5 Fig — This figure lists all factors considered in the paper, but some factors were chosen for further analyses (blue backgrounds). For example, for flight traits, we chose flight style among all four traits from model selection results to interrogate the relationship between flight style and metabolic traits. Statistically significant results are linked by solid lines. Methods that were used for the results are annotated adjacent to the lines. (TIF) [file pbio.3003884.s005.tif]
