# Supplementary material for: Flight style and metabolism shape the tempo of genome evolution in birds
Source: PLoS Biol. 2026 Jul 14;24(7):e3003884. doi: 10.1371/journal.pbio.3003884 (PMC13367715; doi:10.1371/journal.pbio.3003884)
Supplement: S1 Appendix — (DOCX) [file pbio.3003884.s006.docx]

**Supplementary Materials for:**

**Flight style and metabolism shape the tempo of genome evolution in birds**

Yanzhu Ji#, Lei Wu#, Dongming Li, Shaohong Feng, Qi Fang, Ying Xiong, Yongbin Chang, Jacob C. Cooper, Xin Yu, Kai Zhang, Shiyu Tang, Huishang She, Huan Wang, Dezhi Zhang, Gang Song, Ping Fan, Jiaogen Zhou, Liang Ma, Yanhua Qu, Chenxi Jia, Catherine Sheard, J. Andrew DeWoody, Joseph A. Tobias, Guojie Zhang, Weiwei Zhai*, Fumin Lei*

* Corresponding authors:

Weiwei Zhai (weiweizhai@ioz.ac.cn)

Fumin Lei (leifm@ioz.ac.cn)

This document includes:

Supplementary Tables A-M

Table A. Model construction when predicting each of the three evolutionary rates. Note that contig N50 is included in every model and is omitted in the table.

| Model construction | Note |
| --- | --- |
| body mass | Basic models: seven models that only include non-flight trait (i.e., body mass, generation time, and N_e_) |
| gen. time |  |
| N_e_ |  |
| body mass + N_e_ |  |
| gen. time + N_e_ |  |
| body mass + gen. time |  |
| body mass + gen. time + N_e_ |  |
| body mass + one flight trait | Advanced models: inclusion of one flight trait (i.e., flight style, HWI, ALI, or migratory status) to each of the 7 basic models, yielding 28 models in total |
| gen. time + one flight trait |  |
| N_e_ + one flight trait |  |
| body mass + N_e_ + one flight trait |  |
| gen. time + N_e_ + one flight trait |  |
| body mass + gen. time + one flight trait |  |
| body mass + gen. time + N_e_ + one flight trait |  |

Table B. Model selection results of DNA deletion rate, substitution rate, and microsatellite divergence rate (note that contig N50 was omitted in each model for the sake of simplicity). K = the number of estimated parameters for each model; AICc = Akaike information criterion corrected for small sample sizes; ΔAICc = changes in AICc; AICcWt = the Akaike weights; Cum.Wt = cumulative Akaike weights; LL = Log Likelihood values. Best models (ΔAICc < 2) are highlighted in bold, with the significance of each predictor labeled (*, 0.1 < p < 0.5; **, 0.001 < p < 0.01; ***, p < 0.001; ns, non-significant). Instead of showing the result of all 35 models, models with Akaike weights (AICcWt) > 0, or top 10 models are shown.

| Deletion rates (n = 323) |  |  |  |  |  |  |
| --- | --- | --- | --- | --- | --- | --- |
| Model | K | AICc | ΔAICc | AICcWt | Cum.Wt | LL |
| **body mass** + gen. time* + N_e_ + flight style***** | **12** | **-4954.32** | **0** | **0.34** | **0.34** | **2489.66** |
| **body mass** + gen. time* + flight style***** | **11** | **-4954.32** | **0** | **0.34** | **0.68** | **2488.58** |
| **body mass*** + flight style***** | **10** | **-4953.35** | **0.97** | **0.21** | **0.88** | **2487.03** |
| body mass + N_e_ + flight style | 11 | -4952 | 2.33 | 0.11 | 0.99 | 2487.42 |
| gen. time + flight style | 10 | -4946.21 | 8.12 | 0.01 | 1 | 2483.46 |
| Substitution rates (n = 265) |  |  |  |  |  |  |
| Models | K | AICc | ΔAICc | AICcWt | Cum.Wt | LL |
| **body mass*** + N_e_* + flight style***** | **11** | **-1898.96** | **0** | **0.77** | **0.77** | **961** |
| body mass + gen. time + N_e_ + flight style | 12 | -1895.98 | 2.98 | 0.18 | 0.95 | 960.61 |
| body mass + gen. time + flight style | 11 | -1893.48 | 5.48 | 0.05 | 1 | 958.26 |
| Msat. divergence rate (n = 255) |  |  |  |  |  |  |
| Models | K | AICc | ΔAICc | AICcWt | Cum.Wt | LL |
| **body mass**** | **5** | **-4192.69** | **0** | **0.25** | **0.25** | **2101.47** |
| body mass + HWI | 6 | -4190.6 | 2.09 | 0.09 | 0.34 | 2101.47 |
| body mass + N_e_ | 6 | -4190.6 | 2.09 | 0.09 | 0.43 | 2101.47 |
| body mass + gen. time | 6 | -4190.6 | 2.1 | 0.09 | 0.52 | 2101.47 |
| gen. time + migration | 5 | -4190.48 | 2.21 | 0.08 | 0.6 | 2100.36 |
| body mass + migration | 7 | -4189.9 | 2.79 | 0.06 | 0.66 | 2102.18 |
| body mass + N_e_ + HWI | 7 | -4188.49 | 4.2 | 0.03 | 0.7 | 2101.47 |
| body mass + gen. time + HWI | 7 | -4188.49 | 4.21 | 0.03 | 0.73 | 2101.47 |
| body mass + gen. time + N_e_ | 7 | -4188.48 | 4.21 | 0.03 | 0.76 | 2101.47 |
| gen. time + N_e_ | 6 | -4188.43 | 4.26 | 0.03 | 0.79 | 2100.39 |

Table C. *Post hoc* tests for generalized phylogenetic ANCOVA models between DNA deletion rate, generation time, and flight style, showing the comparisons of DNA deletion rates between each two of flight styles after considering body mass. Here, we used abbreviations for flight styles: FS: flapping and soaring; FG: flapping and gliding; CF: continuously flapping; UF: undulating flight; SBF: short burst flight. Statistically significant results are labeled with * (p < 0.05), ** (p < 0.01), or *** (p < 0.001). Significant results that are shared across both datasets are highlighted in bold.

| All species: deletion rate ~ body mass + gen. time + Ne + flight style + contig_N50 | | | | | | |
| --- | --- | --- | --- | --- | --- | --- |
| Category 1 | Category 2 | Estimate | Std. Error | z value | Pr(>z) | Significance |
| FS | Flightless | 9.41E-05 | 4.73E-05 | 1.992 | 0.18 |  |
| FG | Flightless | 5.14E-05 | 4.32E-05 | 1.191 | 0.60 |  |
| CF | Flightless | 8.32E-05 | 3.44E-05 | 2.415 | 0.07 | . |
| UF | Flightless | 9.40E-05 | 4.36E-05 | 2.157 | 0.13 |  |
| SBF | Flightless | 4.61E-04 | 7.58E-05 | 6.088 | <0.001 | *** |
| FG | FS | -4.27E-05 | 3.17E-05 | -1.349 | 1 |  |
| CF | FS | -1.10E-05 | 3.65E-05 | -0.3 | 0.99 |  |
| UF | FS | -1.52E-07 | 4.11E-05 | -0.004 | 0.99 |  |
| SBF | FS | 3.67E-04 | 8.03E-05 | 4.573 | <0.001 | *** |
| CF | FG | 3.17E-05 | 2.91E-05 | 1.092 | 0.66 |  |
| UF | FG | 4.26E-05 | 3.04E-05 | 1.402 | 0.47 |  |
| SBF | FG | 4.10E-04 | 7.68E-05 | 5.334 | <0.001 | *** |
| UF | CF | 1.08E-05 | 2.84E-05 | 0.38 | 0.95 |  |
| SBF | CF | 3.78E-04 | 7.32E-05 | 5.169 | <0.001 | *** |
| SBF | UF | 3.67E-04 | 7.69E-05 | 4.777 | <0.001 | *** |

Table D. *Post hoc* tests for generalized phylogenetic ANCOVA models between substitution rates, body mass (or generation time), and flight style, showing the comparisons of substitution rates between each two of flight styles after considering body mass.

| All species: substitution rate ~ body mass + Ne + flight style + contig N50 | | | | | | |
| --- | --- | --- | --- | --- | --- | --- |
| Category 1 | Category 2 | Estimate | Std. Error | z value | Pr(>z) | Significance |
|  | Estimate | Std. | Error | z | value | Pr(>z) |
| FS | Flightless | -1.46E-05 | 7.39E-03 | -0.002 | 0.99 |  |
| FG | Flightless | 2.77E-03 | 7.39E-03 | 0.375 | 0.93 |  |
| CF | Flightless | 8.01E-03 | 7.32E-03 | 1.095 | 0.60 |  |
| UF | Flightless | 7.74E-03 | 7.44E-03 | 1.04 | 0.63 |  |
| SBF | Flightless | 6.63E-02 | 1.15E-02 | 5.79 | <0.001 | *** |
| FG | FS | 2.78E-03 | 1.22E-03 | 2.287 | 0.08 |  |
| CF | FS | 8.03E-03 | 2.37E-03 | 3.394 | 0.003 | ** |
| UF | FS | 7.75E-03 | 2.28E-03 | 3.407 | 0.003 | ** |
| SBF | FS | 6.63E-02 | 9.19E-03 | 7.221 | <0.001 | *** |
| CF | FG | 5.24E-03 | 2.19E-03 | 2.394 | 0.064 |  |
| UF | FG | 4.97E-03 | 1.96E-03 | 2.54 | 0.04 | * |
| SBF | FG | 6.36E-02 | 9.16E-03 | 6.94 | <0.001 | *** |
| UF | CF | -2.78E-04 | 1.90E-03 | -0.146 | 0.99 |  |
| SBF | CF | 5.83E-02 | 8.99E-03 | 6.487 | <0.001 | *** |

Table E. After excluding flightless birds, the model selection results of DNA deletion rates, substitution rates, and microsatellite divergence rates. Best models (ΔAICc < 2) were highlighted in bold.

| Deletion rates (n = 310) |  |  |  |  |  |  |
| --- | --- | --- | --- | --- | --- | --- |
| Models | K | AICc | Delta_AICc | AICcWt | Cum.Wt | LL |
| **body mass** + gen. time* + flight style*** | **10** | **-4768.54** | **0** | **0.14** | **0.14** | **2394.64** |
| **body mass** + gen. time* + N_e_ (ns) + flight style*** | **11** | **-4768.15** | **0.38** | **0.11** | **0.25** | **2395.52** |
| **body mass** + gen. time* + ALI*** | **8** | **-4768.03** | **0.51** | **0.11** | **0.36** | **2392.25** |
| **body mass*** + flight style*** | **9** | **-4767.7** | **0.84** | **0.09** | **0.45** | **2393.15** |
| **body mass*** + ALI*** | **7** | **-4767.52** | **1.02** | **0.08** | **0.54** | **2390.95** |
| **body mass** + gen. time* + N_e_ (ns) + ALI*** | **9** | **-4766.96** | **1.57** | **0.06** | **0.6** | **2392.78** |
| **body mass** + gen. time (ns) + HWI*** | **7** | **-4766.54** | **1.99** | **0.05** | **0.65** | **2390.46** |
| body mass + N_e_ + flight style | 10 | -4766.17 | 2.37 | 0.04 | 0.69 | 2393.45 |
| body mass + HWI | 6 | -4766.14 | 2.39 | 0.04 | 0.74 | 2389.21 |
| body mass + gen. time + N_e_ + HWI | 8 | -4766.08 | 2.46 | 0.04 | 0.78 | 2391.28 |
| Substitution rates (n = 261) |  |  |  |  |  |  |
| Models | K | AICc | Delta_AICc | AICcWt | Cum.Wt | LL |
| **body mass*** + N_e_ * + flight style***** | **10** | **-1877.79** | **0** | **0.74** | **0.74** | **949.34** |
| body mass + gen. time + flight style | 10 | -1874.35 | 3.44 | 0.13 | 0.87 | 947.61 |
| body mass + gen. time + N_e_ + flight style | 11 | -1874.31 | 3.48 | 0.13 | 1 | 948.69 |
| Msat. div. rates (n = 250) |  |  |  |  |  |  |
| Models | K | AICc | Delta_AICc | AICcWt | Cum.Wt | LL |
| **body mass **** | **5** | **-4109.06** | **0** | **0.25** | **0.25** | **2059.65** |
| body mass + HWI | 6 | -4107.03 | 2.03 | 0.09 | 0.34 | 2059.69 |
| body mass + N_e_ | 6 | -4106.98 | 2.07 | 0.09 | 0.43 | 2059.67 |
| body mass + gen. time | 6 | -4106.96 | 2.1 | 0.09 | 0.52 | 2059.65 |
| body mass + migration | 7 | -4106.38 | 2.68 | 0.07 | 0.59 | 2060.42 |
| gen. time | 5 | -4106.2 | 2.86 | 0.06 | 0.65 | 2058.22 |
| body mass + N_e_ + HWI | 7 | -4104.94 | 4.12 | 0.03 | 0.68 | 2059.7 |
| body mass + gen. time + HWI | 7 | -4104.92 | 4.14 | 0.03 | 0.71 | 2059.69 |
| body mass + gen. time + N_e_ | 7 | -4104.88 | 4.18 | 0.03 | 0.74 | 2059.67 |
| body mass + ALI | 7 | -4104.84 | 4.22 | 0.03 | 0.77 | 2059.65 |

Table F. After removing 10% species with lowest contig N50 scores, model selection results of DNA deletion rate, substitution rate, and microsatellite divergence rate.

| Deletion rates (n = 291) |  |  |  |  |  |  |
| --- | --- | --- | --- | --- | --- | --- |
| Models | K | AICc | Delta_AICc | AICcWt | Cum.Wt | LL |
| **body mass*** + gen. time* + flight style***** | **11** | **-4381.94** | **0** | **0.38** | **0.38** | **2202.44** |
| **body mass*** + flight style***** | **10** | **-4380.87** | **1.08** | **0.22** | **0.61** | **2200.83** |
| body mass + gen. time + N_e_ + flight style | 12 | -4379.87 | 2.07 | 0.14 | 0.74 | 2202.5 |
| body mass + gen. time + ALI | 9 | -4378.78 | 3.16 | 0.08 | 0.82 | 2198.71 |
| body mass + N_e_ + flight style | 11 | -4378.75 | 3.19 | 0.08 | 0.9 | 2200.85 |
| body mass + ALI | 8 | -4377.61 | 4.33 | 0.04 | 0.94 | 2197.06 |
| body mass + gen. time + N_e_ + ALI | 10 | -4376.72 | 5.23 | 0.03 | 0.97 | 2198.75 |
| body mass + N_e_ + ALI | 9 | -4375.53 | 6.41 | 0.02 | 0.99 | 2197.09 |
| gen. time + flight style | 10 | -4374.39 | 7.55 | 0.01 | 1 | 2197.59 |
| Substitution rates (n = 239) |  |  |  |  |  |  |
| Models | K | AICc | Delta_AICc | AICcWt | Cum.Wt | LL |
| **body mass** + gen. time* + N_e_ ** + flight style***** | **12** | **-1721.94** | **0** | **0.76** | **0.76** | **873.66** |
| body mass + N_e_ + flight style | 11 | -1718.3 | 3.64 | 0.12 | 0.89 | 870.73 |
| gen. time + N_e_ + flight style | 11 | -1716.47 | 5.47 | 0.05 | 0.94 | 869.82 |
| body mass + flight style | 10 | -1716.05 | 5.89 | 0.04 | 0.98 | 868.51 |
| body mass + gen. time + flight style | 11 | -1714.93 | 7.01 | 0.02 | 1 | 869.05 |
| Msat. div. rates (n = 230) |  |  |  |  |  |  |
| Models | K | AICc | Delta_AICc | AICcWt | Cum.Wt | LL |
| **gen. time*** | **5** | **-3805.97** | **0** | **0.18** | **0.18** | **1908.12** |
| **body mass*** | **5** | **-3805.68** | **0.29** | **0.16** | **0.33** | **1907.98** |
| **gen. time* + ALI (ns)** | **6** | **-3804.15** | **1.83** | **0.07** | **0.41** | **1908.26** |
| **body mass (ns) + gen. time (ns)** | **6** | **-3804.08** | **1.89** | **0.07** | **0.48** | **1908.23** |
| **N_e_ (ns)** | **5** | **-3803.99** | **1.98** | **0.07** | **0.54** | **1907.13** |
| gen. time + N_e_ | 6 | -3803.88 | 2.09 | 0.06 | 0.6 | 1908.13 |
| body mass + HWI | 6 | -3803.76 | 2.22 | 0.06 | 0.66 | 1908.07 |
| body mass + N_e_ | 6 | -3803.64 | 2.33 | 0.06 | 0.72 | 1908.01 |
| N_e_ + HWI | 6 | -3802.22 | 3.75 | 0.03 | 0.75 | 1907.3 |
| body mass + gen. time + HWI | 7 | -3802.18 | 3.79 | 0.03 | 0.77 | 1908.34 |

Table G. Model selection results using substitution rates derived by Cole et al.

| Models (n = 277) | K | AICc | Delta_AICc | AICcWt | LL |
| --- | --- | --- | --- | --- | --- |
| **body mass** + gen. time* + N_e_ * + flight style**** | **5** | **1072.28** | **0** | **0.49** | **-538.55** |
| **body mass*** + N_e_ (ns) + flight style***** | **5** | **1074.26** | **1.98** | **0.18** | **-541.41** |
| body mass + flight style | 5 | 1074.42 | 2.14 | 0.17 | -552.05 |
| body mass + gen. time + flight style | 6 | 1075.57 | 3.3 | 0.09 | -537.47 |
| gen. time + N_e_ + flight style | 6 | 1077.43 | 5.15 | 0.04 | -535.73 |
| body mass + gen. time + flight style | 6 | 1079.71 | 7.44 | 0.01 | -537.73 |
| gen. time + flight style | 7 | 1080.73 | 8.46 | 0.01 | -532.65 |
| body mass + gen. time + N_e_ + HWI | 7 | 1081.28 | 9 | 0.01 | -538.46 |

Table H. After assigning passerines of “continuously flapping” to “undulating flight”, model selection results of DNA deletion rate, substitution rate, and microsatellite divergence rate.

| deletion rates (n = 323) |  |  |  |  |  |  |
| --- | --- | --- | --- | --- | --- | --- |
| Models | K | AICc | Delta_AICc | AICcWt | Cum.Wt | LL |
| **body mass** + gen. time* + flight style***** | **11** | **-4955.6** | **0** | **0.34** | **0.34** | **2489.23** |
| **body mass*** + gen. time* + N_e_ (ns) + flight style***** | **12** | **-4955.25** | **0.35** | **0.29** | **0.63** | **2490.13** |
| **body mass*** + flight style***** | **10** | **-4954.93** | **0.68** | **0.24** | **0.87** | **2487.82** |
| body mass + N_e_ + flight style | 11 | -4953.39 | 2.22 | 0.11 | 0.99 | 2488.12 |
| gen. time + flight style | 10 | -4948.26 | 7.34 | 0.01 | 0.99 | 2484.48 |
| gen. time + N_e_ + flight style | 11 | -4947.47 | 8.14 | 0.01 | 1 | 2485.16 |
| Substitution rates (n = 304) |  |  |  |  |  |  |
| Models | K | AICc | Delta_AICc | AICcWt | Cum.Wt | LL |
| **body mass*** + N_e_ ** + flight style***** | **11** | **-1900.7** | **0** | **0.81** | **0.81** | **961.87** |
| body mass + gen. time + N_e_ + flight style | 12 | -1897.22 | 3.48 | 0.14 | 0.95 | 961.23 |
| body mass + gen. time + flight style | 11 | -1895.19 | 5.51 | 0.05 | 1 | 959.12 |
| msat. div. rates (n = 255) |  |  |  |  |  |  |
| Models | K | AICc | Delta_AICc | AICcWt | Cum.Wt | LL |
| **body mass**** | **5** | **-4192.69** | **0** | **0.25** | **0.25** | **2101.47** |
| body mass + HWI | 6 | -4190.6 | 2.09 | 0.09 | 0.33 | 2101.47 |
| body mass + N_e_ | 6 | -4190.6 | 2.09 | 0.09 | 0.42 | 2101.47 |
| body mass + gen. time | 6 | -4190.6 | 2.1 | 0.09 | 0.5 | 2101.47 |
| gen. time | 5 | -4190.48 | 2.21 | 0.08 | 0.58 | 2100.36 |
| body mass + migration | 7 | -4189.9 | 2.79 | 0.06 | 0.65 | 2102.18 |
| body mass + N_e_ + HWI | 7 | -4188.49 | 4.2 | 0.03 | 0.68 | 2101.47 |
| body mass + gen. time + HWI | 7 | -4188.49 | 4.21 | 0.03 | 0.71 | 2101.47 |
| body mass + gen. time + N_e_ | 7 | -4188.48 | 4.21 | 0.03 | 0.74 | 2101.47 |
| gen. time + N_e_ | 6 | -4188.43 | 4.26 | 0.03 | 0.76 | 2100.39 |

Table I. Model selection results after grouping flight styles into a 4-category system. We grouped species with “flapping and soaring” and “flapping and gliding” to “soaring and gliding”, and grouped “continuously flapping” and “undulating flight” to “flapping”.

| Deletion rates (n = 323) |  |  |  |  |  |  |
| --- | --- | --- | --- | --- | --- | --- |
| Models | K | AICc | Delta_AICc | AICcWt | Cum.Wt | LL |
| **body mass*** + gen. time* + N_e_ * + flight style***** | **10** | **-4956.45** | **0** | **0.34** | **0.34** | **2488.58** |
| **body mass*** + flight style***** | **8** | **-4955.79** | **0.66** | **0.24** | **0.59** | **2486.12** |
| **body mass** + gen. time (ns) + flight style***** | **9** | **-4955.76** | **0.68** | **0.24** | **0.83** | **2487.17** |
| **body mass*** + N_e_ (ns) + flight style***** | **9** | **-4954.96** | **1.49** | **0.16** | **0.99** | **2486.77** |
| gen. time + N_e_ + flight style | 9 | -4948.29 | 8.16 | 0.01 | 0.99 | 2483.43 |
| gen. time + flight style | 8 | -4948.07 | 8.37 | 0.01 | 1 | 2482.27 |
| Substitution rate (n = 265) |  |  |  |  |  |  |
| Models | K | AICc | Delta_AICc | AICcWt | Cum.Wt | LL |
| **body mass + N_e_ + flight style** | **9** | **-1896.74** | **0** | **0.57** | **0.57** | **957.72** |
| **body mass + gen. time + N_e_ + flight style** | **10** | **-1896.16** | **0.58** | **0.43** | **1** | **958.51** |
| Msat. div. rates (n = 255) |  |  |  |  |  |  |
| Models | K | AICc | Delta_AICc | AICcWt | Cum.Wt | LL |
| **body mass** | **5** | **-4192.69** | **0** | **0.25** | **0.25** | **2101.47** |
| body mass + HWI | 6 | -4190.6 | 2.09 | 0.09 | 0.33 | 2101.47 |
| body mass + N_e_ | 6 | -4190.6 | 2.09 | 0.09 | 0.42 | 2101.47 |
| body mass + gen. time | 6 | -4190.6 | 2.1 | 0.09 | 0.5 | 2101.47 |
| gen. time | 5 | -4190.48 | 2.21 | 0.08 | 0.58 | 2100.36 |
| body mass + migration | 7 | -4189.9 | 2.79 | 0.06 | 0.64 | 2102.18 |
| body mass + N_e_ + HWI | 7 | -4188.49 | 4.2 | 0.03 | 0.67 | 2101.47 |
| body mass + gen. time + HWI | 7 | -4188.49 | 4.21 | 0.03 | 0.7 | 2101.47 |
| body mass + gen. time + N_e_ | 7 | -4188.48 | 4.21 | 0.03 | 0.73 | 2101.47 |
| gen. time + N_e_ | 6 | -4188.43 | 4.26 | 0.03 | 0.76 | 2100.39 |

Table J. Model comparison for fine-graded flight style (6-class) and combined flight style (4-class).

| Deletion rates |  |  |  |  |  |  |
| --- | --- | --- | --- | --- | --- | --- |
| Models | K | AICc | Delta_AICc | AICcWt | Cum.Wt | LL |
| **body mass + gen. time + N_e_ + flight style-4** | **11** | **-4954.68** | **0** | **0.18** | **0.18** | **2488.77** |
| **body mass + gen. time + flight style-4** | **10** | **-4954.36** | **0.32** | **0.15** | **0.33** | **2487.53** |
| **body mass + flight style-4** | **9** | **-4954.35** | **0.33** | **0.15** | **0.48** | **2486.46** |
| **body mass + gen. time + N_e_ + flight style-6** | **12** | **-4954.32** | **0.36** | **0.15** | **0.63** | **2489.66** |
| **body mass + gen. time + flight style-6** | **11** | **-4954.32** | **0.36** | **0.15** | **0.77** | **2488.58** |
| **body mass + flight style-6** | **10** | **-4953.35** | **1.33** | **0.09** | **0.87** | **2487.03** |
| **body mass + N_e_ + flight style-4** | **10** | **-4953.27** | **1.41** | **0.09** | **0.95** | **2486.99** |
| body mass + N_e_ + flight style-6 | 11 | -4952 | 2.69 | 0.05 | 1 | 2487.42 |
| Substitution rates |  |  |  |  |  |  |
| Models | K | AICc | Delta_AICc | AICcWt | Cum.Wt | LL |
| **body mass + N_e_ + flight style-6** | **11** | **-1898.96** | **0** | **0.68** | **0.68** | **961** |
| body mass + gen. time + N_e_ + flight style-6 | 12 | -1895.98 | 2.98 | 0.15 | 0.84 | 960.61 |
| body mass + N_e_ + flight style-4 | 10 | -1894.1 | 4.86 | 0.06 | 0.9 | 957.48 |
| body mass + gen. time + N_e_ + flight style-4 | 11 | -1894 | 4.96 | 0.06 | 0.96 | 958.52 |
| body mass + gen. time + flight style-6 | 11 | -1893.48 | 5.48 | 0.04 | 1 | 958.26 |
| body mass + flight style-6 | 10 | -1873.87 | 25.09 | 0 | 1 | 947.37 |
| body mass + flight style-4 | 9 | -1872.61 | 26.35 | 0 | 1 | 945.66 |
| body mass + gen. time + flight style-4 | 10 | -1870.64 | 28.32 | 0 | 1 | 945.75 |

Table K. Model selection results after divergence rate is incorporated into the models.

| Deletion rates |  |  |  |  |  |
| --- | --- | --- | --- | --- | --- |
| Models (n = 323) | K | AICc | Delta_AICc | AICcWt | LL |
| **body mass*** + gen. time* + div. rate** + N_e_ (ns) + flight style***** | **5** | **-4960.11** | **0** | **0.37** | **2468.61** |
| **body mass*** + gen. time* + div. rate** + flight style***** | **5** | **-4959.91** | **0.204** | **0.34** | **2462.88** |
| **body mass*** + div. rate** + flight style***** | **5** | **-4958.26** | **1.857** | **0.15** | **2449.42** |
| body mass + div. rate + N_e_ + flight style | 5 | -4956.93 | 3.187 | 0.08 | 2450.63 |
| body mass + gen. time + N_e_ + flight style | 6 | -4954.32 | 5.79 | 0.02 | 2469.44 |
| body mass + gen. time + flight style | 6 | -4954.32 | 5.793 | 0.02 | 2472.15 |
| body mass + flight style | 6 | -4953.35 | 6.762 | 0.01 | 2468.64 |
| body mass + N_e_ + flight style | 6 | -4952 | 8.117 | 0.01 | 2466.85 |
| gen. time + div. rate + flight style | 6 | -4951.87 | 8.246 | 0.01 | 2462.91 |
| gen. time + div. rate + N_e_ + flight style | 6 | -4951.62 | 8.491 | 0.01 | 2452.6 |
| Substitution rates |  |  |  |  |  |
| Models (n = 265) | K | AICc | Delta_AICc | AICcWt | LogLik |
| **body mass*** + div. rate*** + flight style***** | **5** | **-1877.82** | **0** | **0.37** | **916.63** |
| **body mass** + gen. time (ns) + div. rate*** + flight style***** | **5** | **-1877.53** | **0.288** | **0.32** | **912.5** |
| **body mass** + gen. time (ns) + div. rate*** + N_e_ (ns) + flight style***** | **5** | **-1876.17** | **1.649** | **0.16** | **902.15** |
| body mass + div. rate + N_e_ + flight style | 5 | -1875.72 | 2.098 | 0.13 | 901.54 |
| gen. time + div. rate + flight style | 6 | -1869.83 | 7.989 | 0.01 | 917.75 |
| Msat. div. rates |  |  |  |  |  |
| Models (n = 255) | K | AICc | Delta_AICc | AICcWt | LogLik |
| **body mass*** | **5** | **-4192.69** | **0** | **0.17** | **2101.47** |
| **body mass* + div. rate (ns)** | **5** | **-4191.26** | **1.44** | **0.08** | **2100.36** |
| body mass + HWI | 5 | -4190.6 | 2.09 | 0.06 | 2098.77 |
| body mass + N_e_ | 5 | -4190.6 | 2.09 | 0.06 | 2099.2 |
| body mass + gen. time | 6 | -4190.6 | 2.1 | 0.06 | 2101.47 |
| gen. time | 6 | -4190.48 | 2.21 | 0.05 | 2101.8 |
| body mass + migratory status | 6 | -4189.9 | 2.79 | 0.04 | 2101.47 |
| body mass + div. rate + HWI | 6 | -4189.16 | 3.53 | 0.03 | 2100.68 |
| body mass + gen. time + div. rate | 6 | -4189.15 | 3.55 | 0.03 | 2100.39 |
| body mass + div. rate + N_e_ | 6 | -4189.15 | 3.55 | 0.03 | 2099.45 |

Table L. The phylogenetic ANCOVA and PGLS results when regressing FMR, Heart Mass, or family-level FER against body mass and flight style. Note that only the results of flight style were shown, and the correlations with body mass were omitted in this table. Significant results (p < 0.05) were highlighted in bold.

| Flight style | numDF | F-value | p-value |
| --- | --- | --- | --- |
| FMR (n = 107) | 5 | 1.44 | 0.22 |
| Heart Mass (n = 97) | 5 | 3.48 | **0.006** |
| Flight Efficiency Ratio (family-level, n = 33) | 5 | 7.00 | **0.0003** |

# Table M. Regression results between residuals of heart mass (or FER) and each of the three evolutionary rates. Statistically significant (p ≤ 0.05, one-tailed tests) results are highlighted in bold.

| Response variable: deletion rates |  |  |  |  |
| --- | --- | --- | --- | --- |
| Predictor | Estimate | Std. Error | T value | P value |
| **Residuals of heart mass** | **-8.01E-05** | **4.90E-05** | **-1.64** | **0.05** |
| **Residuals of FER** | **-6.08E-05** | **3.67E-05** | **-1.66** | **0.05** |
| Response variable: substitution rates |  |  |  |  |
| Predictor | Estimate | Std. Error | T value | P value |
| Residuals of heart mass | -4.97E-03 | 4.99E-03 | -1.00 | 0.32 |
| Residuals of FER | 2.36E-03 | 1.30E-03 | 1.82 | 0.96 |
| Response variable: msat. div. rates |  |  |  |  |
| Predictor | Estimate | Std. Error | T value | P value |
| Residuals of heart mass | 1.45E-05 | 2.41E-05 | 0.60 | 0.55 |
| Residuals of FER | 3.70E-05 | 1.50E-05 | 2.47 | 0.98 |
