# Supplementary material for: Flight style and metabolism shape the tempo of genome evolution in birds
Source: PLoS Biol. 2026 Jul 14;24(7):e3003884. doi: 10.1371/journal.pbio.3003884 (PMC13367715; doi:10.1371/journal.pbio.3003884)
Supplement: S1 Text — (DOCX) [file pbio.3003884.s007.docx]

**S1 Text**

1. Deletion length calculation

In this manuscript, we updated the method to calculate deletion length compared to our preceding work (Ji et al., 2022). Specifically, we calculated deletion length using ancestral (“root”) sequence as the reference, thus enabled the inclusion of all avian species. Our updated deletion length largely agreed with our previous results (Figure 1a). Additionally, since the calculation was based on a certain number (i.e., 100,000) of 1-kb alignment blocks, we determined if the number of blocks used was sufficient to calculate deletion length, by first extracting 300,000 1-kb alignment blocks and then subsampling from these for a range of block numbers (from 10,000 – 100,000) repeatedly. Finally, we found our sample of 100,000 blocks sufficient to calculate deletion length, since the variance tend to decrease as the number of blocks increase (Figure 1B, 1C).


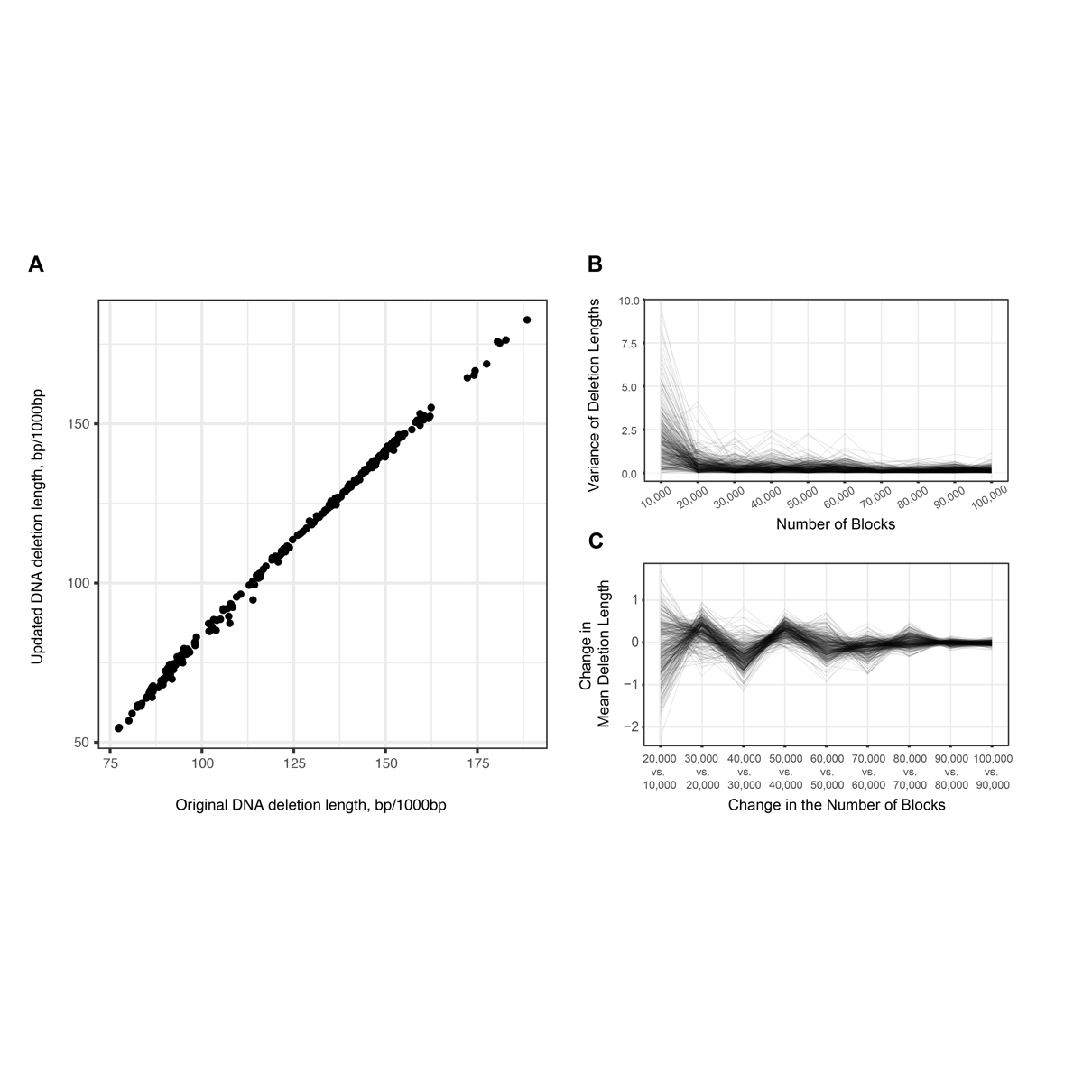


Figure 1. (A) Justification of our updated method when measuring DNA deletion length. The correlation between original DNA deletion length (x-axis) and the updated DNA deletion length (y-axis). Our updated method covered deletion length of more species but with highly correlated results when compared to the previous estimate. (B-C) Justification of the choice of block number when measuring DNA deletion length, showing (B) The variance of deletion length and (C) the change of mean deletion length when comparing deletion lengths measured using different number of blocks. The values for each number of blocks were calculated using 3 replicates per species, sampled without replacement from 300,000 blocks in total. The variance of deletion length and change in mean deletion length of each species are connected with lines.

2. Scoring Aerial Lifestyle Index (ALI)

Aerial Lifestyle Index, ranging from “0” to “3”, measures the use of flight during foraging behavior and daily routines. Aerial lifestyle category of “0” refers to completely flightless birds, whereas category “3” describes highly aerial species, which flight is an integral part of the species’ daily routine, such as hummingbirds, swifts and swallow, and soaring birds. Categories “1” and “2” refer to infrequent and moderate fliers, respectively (Weeks et al., 2022).

3. Impact of flightless birds on modeling results

We found that the trend between HWI and residuals of deletion rates (or substitution rates) were different for flightless birds, as demonstrated by the scatterplot between the residuals of deletion rates and substitution rates (both after correcting for body mass) and HWI (Figure 2). Whereas the birds excluding flightless birds showed a negative correlation between HWI and both residuals, flightless birds have the lowest HWI but not the highest residuals (Figure 2). Therefore, we ran the model selection after removing flightless birds.


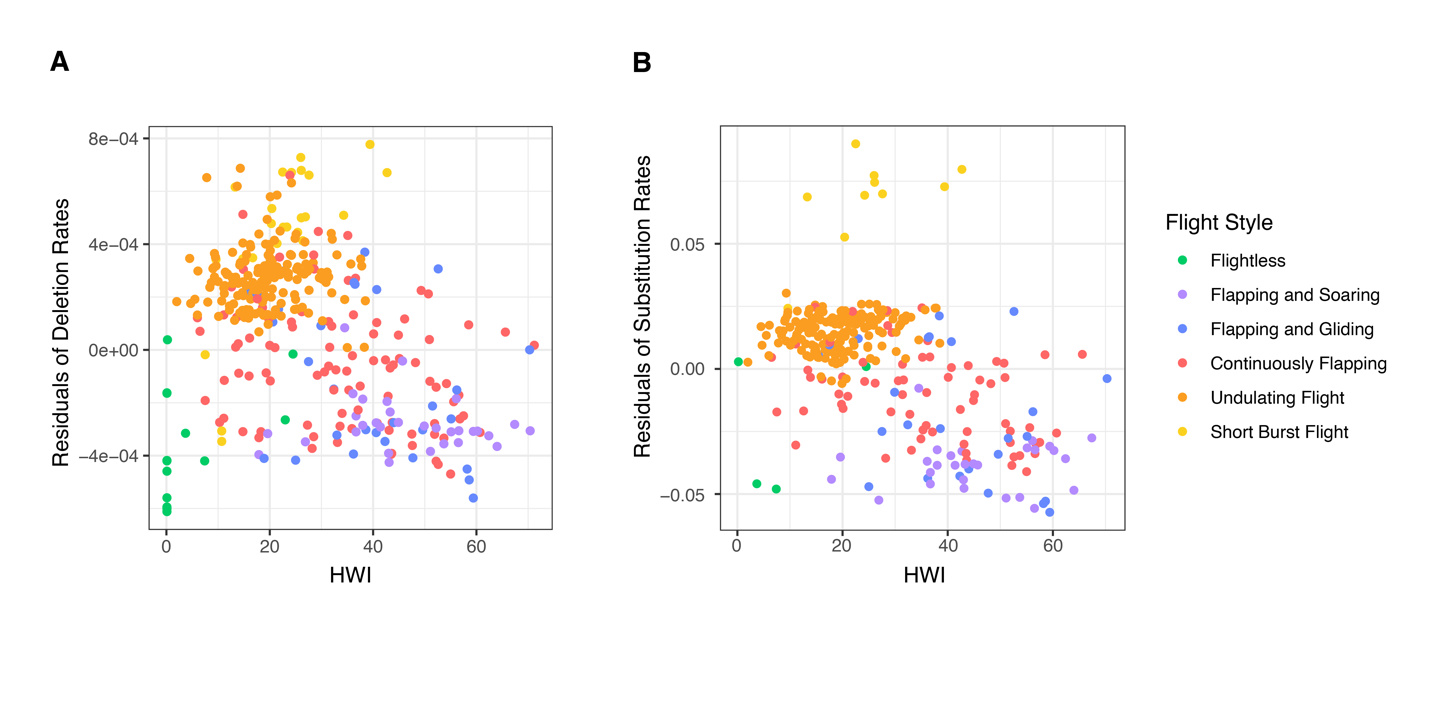


Figure 2. Scatterplot between HWI and residuals of (A) deletion rates, and (B) substitution rates, showing that trend between these variables are different for flightless birds and other birds.

4. Birds with weak flight ability (as indicated by aerial lifestyle) have elevated substitution rates in mitochondrial genes

Since the substitution rate we compiled from Cole et al., 2022 consisted of mostly genomic DNA, it is unclear whether mitochondrial substitution rates calculated using a similar approach would yield consistent result as nuclear genomic substitution rates. Therefore, we subsequently calculated mitochondrial substitution rates based on 4-fold degeneration sites in mt. genes (including ND1, ND2, ND3, ND4, ND5, COX1, COX2, COX3, CYTB, and ATP6) since there were very few intergenic regions. To mirror the method of deriving deletion rates and substitution rates, we first reconstructed ancestral sequence of avian mt. genes by building multiple sequence alignment of mitochondrial genes including two outgroup species (*Crocodylus porosus* and *Anolis puctatus*) with Progressive Cactus (Armstrong et al., 2020). Next, 4-fold degeneration sites were extracted from the resulting alignments and substitution rates were calculated by dividing substitutions (corrected for multiple hits) by divergence time.

Among the models we previously constructed, we found mitochondrial substitution rates were best predicted by aerial lifestyle index with the combination of generation time or body mass (Table), with an increasing trend for flighted birds from aerial lifestyle index “3” (most aerial) to “1” (least aerial; Figure 3).

Table. Model selection results of mitochondrial substitution rates.

|  | K | AICc | ΔAICc | AICcWt | Cum.Wt | LL |
| --- | --- | --- | --- | --- | --- | --- |
| **gen. time + ALI** | **7** | **-3602.23** | **0** | **0.51** | **0.51** | **1808.3** |
| **body mass + ALI** | **7** | **-3601.6** | **0.63** | **0.38** | **0.89** | **1807.98** |
| gen. time + flight style | 9 | -3598.18 | 4.05 | 0.07 | 0.96 | 1808.39 |
| body mass + flight style | 9 | -3597.2 | 5.03 | 0.04 | 1 | 1807.9 |
| gen. time | 4 | -3585.81 | 16.42 | 0 | 1 | 1796.97 |
| body mass | 4 | -3585.21 | 17.02 | 0 | 1 | 1796.67 |
| gen. time + migration | 6 | -3583.98 | 18.25 | 0 | 1 | 1798.13 |
| gen. time + HWI | 5 | -3583.88 | 18.35 | 0 | 1 | 1797.04 |
| body mass + gen. time | 5 | -3583.81 | 18.42 | 0 | 1 | 1797 |
| body mass + migration | 6 | -3583.45 | 18.78 | 0 | 1 | 1797.86 |
| body mass + HWI | 5 | -3583.33 | 18.9 | 0 | 1 | 1796.77 |


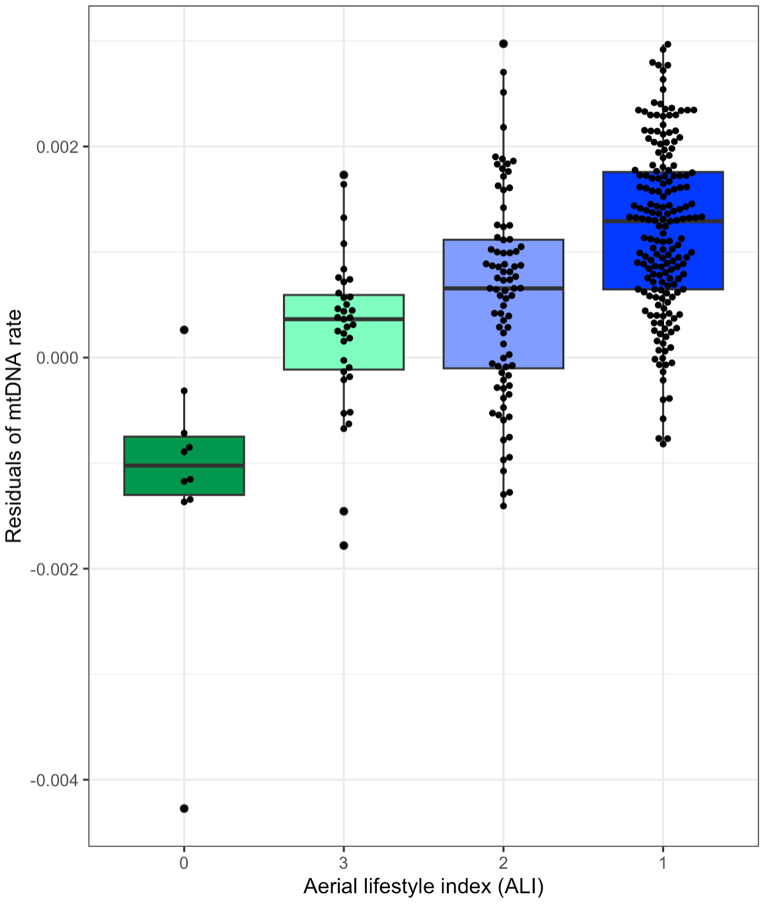


Figure 3. Residuals of mitochondrial substitution rates after considering generation time. For flighted birds, there is an increasing trend of mitochondrial substitution rates from aerial lifestyle index “3” to “1”.

References

Armstrong, J., Hickey, G., Diekhans, M., Fiddes, I. T., Novak, A. M., Deran, A., Fang, Q., Xie, D., Feng, S., Stiller, J., Genereux, D., Johnson, J., Marinescu, V. D., Alföldi, J., Harris, R. S., Lindblad-Toh, K., Haussler, D., Karlsson, E., Jarvis, E. D., … Paten, B. (2020). Progressive Cactus is a multiple-genome aligner for the thousand-genome era. *Nature*, *587*(7833), 246–251. https://doi.org/10.1038/s41586-020-2871-y

Cole, T. L., Zhou, C., Fang, M., Pan, H., Ksepka, D. T., Fiddaman, S. R., Emerling, C. A., Thomas, D. B., Bi, X., Fang, Q., Ellegaard, M. R., Feng, S., Smith, A. L., Heath, T. A., Tennyson, A. J. D., Borboroglu, P. G., Wood, J. R., Hadden, P. W., Grosser, S., … Zhang, G. (2022). Genomic insights into the secondary aquatic transition of penguins. *Nature Communications*, *13*(1), 3912. https://doi.org/10.1038/s41467-022-31508-9

Ji, Y., Feng, S., Wu, L., Fang, Q., Brüniche-Olsen, A., DeWoody, J. A., Cheng, Y., Zhang, D., Hao, Y., Song, G., Qu, Y., Suh, A., Zhang, G., Hackett, S. J., & Lei, F. (2022). Orthologous microsatellites, transposable elements, and DNA deletions correlate with generation time and body mass in neoavian birds. *Science Advances*, *8*(35), 31.

Weeks, B. C., O’Brien, B. K., Chu, J. J., Claramunt, S., Sheard, C., & Tobias, J. A. (2022). Morphological adaptations linked to flight efficiency and aerial lifestyle determine natal dispersal distance in birds. *Functional Ecology*, *36*(7), 1681–1689. https://doi.org/10.1111/1365-2435.14056
