# Supplementary material for: Flight style and metabolism shape the tempo of genome evolution in birds
Source: PLoS Biol. 2026 Jul 14;24(7):e3003884. doi: 10.1371/journal.pbio.3003884 (PMC13367715; doi:10.1371/journal.pbio.3003884)
Supplement: Attachment — Submitted filename: Comments_PB_final.pdf [file pbio.3003884.s009.pdf]

Reviewer #1: In this study the authors consider how various ecological and life history traits effect rates of evolution in birds. The results of the study are intriguing, and it is overall an excellent study. I only have a few suggestions.

We appreciate the reviewer for the insightful summary and comments. We have addressed the reviewer's comments in great detail below, and we are quite grateful that these changes have significantly strengthened the manuscript.

Q1.1 First off, I would strongly suggest including a table that give readers an idea of the relative influence of life history traits vs the other traits that the authors consider, since the current paradigm is that substitution rates are determined largely by generation time. For example, my interpretation of Fig. 2 A-C would be that most of the relationship between body size and substitution rates is driven by larger species having longer generation time.

Reply: we thank the reviewer for the helpful suggestion. Following your advice, we have added a table (Table 1) and a figure (supplementary Figure 2H) to illustrate the contribution of each trait to deletion rates and substitution rates (see attached below). As shown, for deletion rates, generation time and effective population size (N_e) explained relatively smaller part of the variation of deletion rates, while body mass and flight style explained much of the variation. For substitution rates, flight style, body mass, and N_e all explained significant percentages of the variation (generation time is not included in the best model). We hope the decomposition of variation by partial R^2 has made it clear.

Table 1. Partial R^2 values of predictors that explain deletion rates and substitution rates as determined by best models.

Full model: deletion rate ~ body mass + gen. time + N_e + flight style	
Predictor	Partial R^2
Body mass	0.033
Gen. time	0.014
N_e	0.007
Flight style	0.116
**Full model: substitution rate ~ body mass + N_e + flight style**	
Predictor	Partial R^2
Body mass	0.077
N_e	0.098
Flight style	0.19

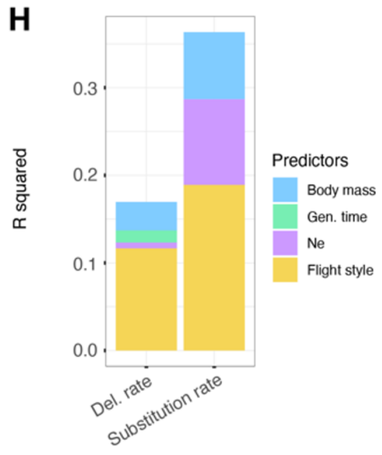

Supplementary Figure 2H. Partial R^2 values for the predictors in best models for deletion rates and substitution rates.

Q1.2. Another thing that surprised me was that the authors did not seemingly consider the potential influence of effective population size on rates of substitution. For example a fairly recent high profile study (Bergeron et al. 2023) showed evidence that this factor influences rates of evolution across all vertebrates. Stiller et al (2024), which the authors cite and includes at least one shared author that I spotted (and I assume I am missing a few more!) also calculated effective population size across birds, so it seems that it would be relatively straightforward to include it. At the very least, Bergeron should be cited and the authors should make it clear why they didn't include it. On the other hand, if that is one of the "life history traits" that is included in the model only shown in the supplement, my first comment goes double!

Reply: we appreciate the reviewer's suggestion about effective population size (N_e). The reviewer is correct that N_e could have influenced the rate of molecular evolution by the "drift-barrier hypothesis". In the original analysis, we didn't include it partly due to the fact that N_e is an "intermediate trait" between macro phenotypes (e.g. generation time) and molecular phenotypes (e.g. substitution rates). It could be correlated with both macro and micro phenotypes which will increase the overall multi-collinearity of the model and can obstruct the interpretability of the model.

Following your advice, we retrieved N_e from B10K consortium (Germain et al. 2023), we found the collinearity between N_e and body mass or generation time is not as severe (Variance Inflation Factor < 2). Therefore, we included N_e into our analysis by incorporating N_e into our latest results. Specifically, we redesigned models and re-performed model selection after expanding the 11-model set into a 35-model set with N_e (for model construction, see supplementary Table 1). Our updated results stay consistent with our previous findings, in that models with flight characters are always among the best models for substitution rates and deletion rates, but not for microsatellite divergence rates (supplementary Table 2). The updated results also showed that N_e is among the best models that predict substitution rates, but not the other two rates (supplementary Table 2). The rest results of regression models also remained largely unchanged.

We thank the reviewer for recommending N_e into the model and this analysis indeed increase the connection of our findings with the community.

Q1.3 Another factor that one might expect to be related to rates of evolution is diversification rate. In fact, there is a major debate about whether diversification rates drive variation in rates of morphological trait evolution (e.g., Adams et al. 2009 vs Rabosky et al. 2013). I think there would be some reader interest in knowing what is going on with diversification rates and rates of molecular evolution.

If the authors wanted to include this, tip level metrics of diversification rate are easily to calculate, and would be straightforward to incorporate into their species level modelling framework. See, for example, the method used in the classic Jetz et al. (2012) study, which also has a tree they could use to calculate accurate rates (since a tree that doesn't include all birds might give incorrect rates).

Reply: we appreciate the reviewer for the interesting suggestion. Indeed, as the reviewer said, diversification rates have been related to the rates of morphological changes, and therefore it is possible that diversification rates are also related to molecular evolutionary rates.

To calculate diversification rates, we used the same method as the reviewer suggested (in Jetz et al.). To account for the uncertainties in Jetz et al's trees, the rates are first calculated using 100 full trees constructed using the Hackett backbone. For each species, diversification rates are then averaged using the medians across the results of 100 trees. Next, we added diversification rates to the modeling framework. After running the model selection that including diversification rates, we found diversification rates are among the best models that predict deletion rates and substitution rates, but not microsatellite divergence rates. Flight style contributed to the best models similar to the results without diversification rates (supplementary Table 11).

We have incorporated the modeling selection with diversification rates as part of the "sensitivity analyses" in Results, Lines (443-450) as well as Material and Methods (Lines 289-302).

Q1.4 Another small quibble is that the authors did not make it clear that they adjusted their PGLS analysis for the amount of phylogenetic signal observed in uncorrected model residuals. Since the authors used nlme for this analysis, I assumed that they used the corBrownian function to do just that. However, readers not intimately familiar with that R package might not realize it.

A brief note "using the corBrownian function to adjust each model for the amount of phylogenetic signal observed in residuals" around line 210 is all that is needed.

Reply: We appreciate the rigorous thought from the reviewer. You are quite right that in PGLS models we need to specify the correlation structure used to correct for the residuals. When fitting the PGLS models, we used corPagel to fit lambda and the regression model at the same time. This is more flexible compared to corBrownian structure, which uses a default lambda value of 1 when fitting the models.

We have now added the detailed information in Material and Methods (Lines 254-256).

Q1.5 Finally, in the discussion the authors may want to reference the metabolic theory of ecology (Brown 2004, Sibley et al. 2012), since their results broadly agree with it. Even though the authors did not attempt to specifically evaluate whether quarter power scaling was present, this is one of the clearest demonstrations that I am aware of that metabolic rates influence sequence substitution rates even after accounting for life history variation. I think that crowd will be excited by the result.

Reply: we thank the reviewer for this insightful recommendation. The reviewer has suggested the Metabolic Theory of Ecology could help to contextualize our main finding. Our results indeed fall into the prediction of the Metabolic Theory of Ecology, and revealed a previously undescribed aspect between flight traits (which we think closely reflect metabolic efficiency) and certain genomic evolutionary rates. We have added one paragraph in the discussion (Lines 557-575) to reflect these changes.

All that said, in case it is not clear at this point, let me say again that this is an excellent analysis. I have no issues with the conclusions that the authors have drawn, or the methods that they chose to use.

We thank the reviewer for the encouraging comments of our work!

Cited:

Adams, D. C., Berns, C. M., Kozak, K. H., & Wiens, J. J. (2009). Are rates of species diversification correlated with rates of morphological evolution?. Proceedings of the Royal Society B: Biological Sciences, 276(1668), 2729-2738.

Bergeron, L. A., Besenbacher, S., Zheng, J., Li, P., Bertelsen, M. F., Quintard, B., ... & Zhang, G. (2023). Evolution of the germline mutation rate across vertebrates. Nature, 615(7951), 285-291.

Brown, J. H., Gillooly, J. F., Allen, A. P., Savage, V. M., & West, G. B. (2004). Toward a metabolic theory of ecology. Ecology, 85(7), 1771-1789.

Jetz, W., Thomas, G. H., Joy, J. B., Hartmann, K., & Mooers, A. O. (2012). The global diversity of birds in space and time. Nature, 491(7424), 444-448.

Rabosky, D. L., Santini, F., Eastman, J., Smith, S. A., Sidlauskas, B., Chang, J., & Alfaro, M. E. (2013). Rates of speciation and morphological evolution are correlated across the largest vertebrate radiation. Nature communications, 4(1), 1958.

Sibly, R. M., Brown, J. H., & Kodric-Brown, A. (Eds.). (2012). Metabolic ecology: a scaling approach. John Wiley & Sons.

Stiller, J., Feng, S., Chowdhury, A. A., Rivas-González, I., Duchêne, D. A., Fang, Q., ... & Zhang, G. (2024). Complexity of avian evolution revealed by family-level genomes. Nature, 629(8013), 851-860.

We appreciate the list of references recommended by the reviewer. We have cited them in our latest version. Thank you!

Reviewer #2: As data on molecular rates becomes more available, there is an increasing field that examines the predictors of these rates using comparative data. Several papers have considered flight as a potential predictor of evolutionary rates, due to its potential connections with metabolism. This paper adds to that literature. The results are potentially interesting but I see several areas where improvement or further examination is needed:

We thank the reviewer for the encouraging comments. Below we have addressed the individual comments in great detail.

Q2.1 First, the study uses AIC model comparison with PGLS models, using the gls function. It is important when doing this that the maximum likelihood estimator for the variance is set to "ML" and not the default "REML" for this function, which results in models that cannot be compared. The paper does not specify if this was done, and I could not access the zenodo link to check the code myself. Please confirm and clarify.

Reply: we thank the reviewer for the rigorous thought. The reviewer pointed out an important detail when using AIC model comparison with PGLS models. It is necessary to use “ML” because we compare models with different fixed-effect structures using AIC, which requires likelihoods to be computed under ML for valid comparison.

We fully agree with the reviewer and have the same intuition as the reviewer. To be more precise, we indeed used "ML" when fitting PGLS models, and has added the specification in the methods part (Lines 256). Additionally, we have made sure that our code is accessible (https://zenodo.org/records/20093624?preview=1&token=eyJhbGciOiJIUzUxMiJ9.eyJpZCI6IjM1MjI2NmM4LWUwOWMtNDU5ZS05YTgwLWRiNjkyNDE5NWIxNiIsImRhdGEiOnt9LCJyYW5kb20iOiIyMDk2MDBiMzdkYmFhNGQzMTk2ODMyODdmYjBjMDU5MyJ9.sxu7ozP5uSFGQszBjuy9vE4_sfxxuJcH6FQh0r8hUz-gSqVer-HIea0ZK4TyYrZelzG_9YJXeuq3h47ZNamnFQ).

We appreciate the reviewer for the very kind reminder. This is indeed an important point for this type of analysis.

Q2.2 Second, the Materials and Methods section is written in a very confusing way. I think that what happened is that the authors wrote the paper for a journal such as PNAS in which the Results and Discussion come before the Methods, but then they moved the Methods before the results for this submission without editing it carefully to include the necessary information in Methods before the Results section. Consequently, there were several areas where they did not include important information in the Methods section that was later presented in Results. This needs to be reorganized for readability.

Reply: we appreciate the reviewer's very insightful observation. The reviewer was correct that our manuscript was indeed written for another journal where Results and Discussion come before the Methods. To make sure that important information comes up first in Methods rather than in Results, we have thoroughly reorganized the Methods and the Results part. Specifically:
(1) in Methods section "Calculation of genomic evolutionary distances and rates", we have added information that describe data acquisition and species sampling (Lines 124 – 133).
(2) in Methods section "Scoring and compiling of phenotypic traits", we added details about trait collection and flight style scoring (Lines 195 – 210).
(3) after Methods section "Model building and Model selection", we supplemented another section "Sensitivity analyses" to cover what we did to test the robustness of our results (Lines 271 – 302).
(4) in Method section "Correlation between energy metabolism, flight characters, and genomic evolutionary rates", we emphasized why we collected these metabolic metrics (Lines 306 – 314).

We have rechecked the manuscript such that necessary information is provided first. We hope these changes help make the manuscript flow better than the original version.

Q2.3 Third, I found the flight style designations confusing. I am surprised that so many passerines were classified as undulating rather than continuous flight -- this does not seem right to me. I also was not sure what flapping and gliding meant. I could not easily find a list of species and their category, so this was hard to evaluate. Given that there differences in the evolutionary rates for undulating but not continuous flight, this needs critical examination, and probably fewer categories are better. Additionally, I found that the addition of the Aerial Lifestyle Index did not add anything useful to the paper, and probably just added an extra layer of complexity that made the results harder to interpret. I would recommend removing this.

Reply: we appreciate the critical comments from the reviewer. Given that several important issues were raised, we would like to break them down and address them one by one carefully.

1. Clarification of flight style designations and the distinction between "undulating flight" and "flapping and gliding"

It could be the case the original manuscript did not define these flight styles clearly enough. To strengthen this, we have: (1) edited the definition of flight styles in the Methods section; (2) included a supplementary data listing all species and their assigned flight style category, so that our classification can easily accessed.

Regarding the reviewer's comment that many passerines were classified as "undulating flight": This classification follows the established literature (Pennycuick 2008; Bruderer et al. 2010; Vincze et al. 2018), where "passerine-type flight" (our "undulating") is defined by intermittent wing-bounding (wings folded against body), even if the bounding is subtle in some species. Many passerines do briefly fold their wings against the body during the non-flapping phase. If the bounding phase is short, the flight trajectory may not be that "wavy" thus making the bounding phase hard to notice. This is distinct from continuous flapping (e.g., hummingbirds, some shorebirds), which involves no regular non-flapping phases. It is also distinguished from "flapping and gliding", in which the non-flapping gliding phase is much longer. We have now clarified this distinction in the text (Lines 214 – 224).

We also acknowledge that some species may show intermediate behavior. Following the reviewer's recommendation to regroup some categories, we did perform additional test for checking the robustness of the results (see next section). We hope the newly included table and clearer definitions will resolve the confusion.

For consistency with existing literature, we retained the original assignment for the majority of analysis.

2. Grouping of flight style categories
The reviewer suggested that fewer flight categories may help with the situation here. We agree that practically these flight styles sometimes have very subtle differences. Therefore, we re-ran the model selection after grouping these flight styles into a four-category system: we grouped birds with "flapping and soaring" and "flapping and gliding" into "soaring and gliding", and grouped "continuously flapping" and "undulating flight" into "flapping". The model selection result was consistent with the original results (see supplementary Table 10).

3. Removal of the Aerial Lifestyle Index
The reviewer raised a concern that ALI may add complexity without contributing meaningfully to the main findings. We included it at first because (1) ALI reflects the aerial behavior of birds which may relate to their metabolic demand, and (2) collaborators provided us this manually scored data. To address the reviewer's concern, we have moved the result about ALI to the supplement and reduced the mention of ALI in the manuscript, including:

(1) Moved the definition of ALI to the supplementary text (main Methods now only notes its existence).
(2) Deleted any mention of ALI from the main Results, except for one sentence acknowledging that it appeared in one best model (with a reference to supplementary Table 6, Lines 395 – 398).

We appreciate the reviewer for the rigorous thoughts and suggestions.

Q2.4 Fourth, the paper rests on an estimate of evolutionary rates that is a root to tip distance based on an ancestral state reconstruction. But, of course, the ancestral state estimation could be affected by heterogeneity in evolutionary rates. How this kind of circularity could affect the estimation of rates is not clear to me, but I think it deserves mention and discussion.

Reply: we thank the reviewer for raising this important point. We agree that the rates calculated by root-to-tip distances are inherently affected by ancestral sequence reconstruction, which could be affected by rate heterogeneity (or any model of rate evolution used in ancestral sequence reconstruction). We also agree it might sound a bit "circular", where we first estimate ancestral sequence based on an evolutionary model/process, then subsequently re-estimate the rate between ancestral and extent sequences. We used this approach mainly because it is not yet possible to derive the rate of evolution for deletion and microsatellite rates from the evolutionary trees. However, we still would like to calculate the rates (substitution, microsatellite and deletion rates) using the same framework. Thus, we employed the approach (i.e. root-to-tip distance) we took.

To evaluate whether the circularity affects our conclusions, we used the phylogeny-based substitution rate from Cole et al. (which avoided ancestral reconstruction for rate estimation). We then compared model selection results using these branch-length rates versus our root-to-tip substitution rates. The outcomes were very similar (see Supplementary Table 7), and all key conclusions remained unchanged.

Given the above observations, for the majority of our analyses, we chose to use the root-to-tip method because it can be applied to all three rates. we have presented the alternative result for substitution rate in Methods (Lines 274-278), in Results (lines 399-402), and in Supplementary Table 7.

Q2.5 Finally, I thought the paper did a rather poor job of explaining WHY differences in flight style might result in differences in evolutionary rate. Why should we see higher rates in undulating flight species but not continuously flapping? (e.g., Figure 2). If you are going to make these categorizations, then there should be an hypothesis or predictions for how and why the variation should affect the outcome variable, and I don't see that here. I can imagine metabolic differences between, say, flapping and soaring species, but the finer-scale designations among flapping species are much more tenuously connected to metabolism, if at all. This also relates to my third comment above. As such, the results are difficult to connect to biology.

Reply: we want to thank the reviewer for the critical comments. The reviewer asked whether we could present a clear biological hypothesis linking finer-scale flight style categories (e.g., undulating flight vs continuously flapping) to evolutionary rates, rather than documenting an empirical pattern.

While we agree that we aim to stay "parsimonious" in terms of scoring categorical variables, we employed the finer classification system because birds in these finer categories are different in terms of energy metabolism and efficiency. Our hypotheses include: for birds with short burst flight, previous studies suggest that they have relatively smaller hearts and could get exhausted quickly (Altimiras et al. 2017; Nespolo et al. 2018). These physical limitations could lead to higher oxidative stress. For birds with undulating flight, their round-shaped wings and relatively low energy efficiency during flight predict that they may have elevated metabolic rates (Bishop 2005; Guigueno et al. 2019). Although contradictory evidence exists (Vincze et al. 2018), there is consistent evidence that it involves distinct metabolic profiles. We have now updated these into our introduction (Lines 85-92).

Additionally, to test whether the fine-grained classification captures meaningful biological variation, we compared the two flight style scoring systems via model comparison. The model comparison results show that for deletion rates, the two flight style systems performed equally. For substitution rates, the fine-graded flight style system performed better (supplementary Table 10). Therefore, the fine-graded flight system explains the data best, supporting our hypothesis that these flight styles are different.

We have added the literature suggesting fine-graded flight style system in the introduction (Lines 85-92). We have added the model selection in Methods (Lines 284-286) and in Results (Lines 437-441). We hope this new result help addresses the reviewer's concern.

Citations:

Altimiras J, Lindgren I, Giraldo-Deck LM, Matthei A, Garitano-Zavala Á. 2017. Aerobic performance in tinamous is limited by their small heart. A novel hypothesis in the evolution of avian flight. *Sci. Rep.* 7:15964.

Burgomaster KA, Hughes SC, Heigenhauser GJF, Bradwell SN, Gibala MJ. 2005. Six sessions of sprint interval training increases muscle oxidative potential and cycle endurance capacity in humans. *J. Appl. Physiol.* 98:1985–1990.

Germain RR, Feng S, Chen G, Graves GR, Tobias JA, Rahbek C, Lei F, Fjeldså J, Hosner PA, Gilbert MTP, et al. 2023. Species-specific traits mediate avian demographic responses under past climate change. *Nat. Ecol. Evol.* 7:862–872.

Gibala MJ, McGee SL. 2008. Metabolic adaptations to short-term high-Intensity interval training. *Exerc. Sport Sci. Rev.* 36:58–63.

Guigueno MF, Shoji A, Elliott KH, Aris-Brosou S. 2019. Flight costs in volant vertebrates: A phylogenetically-controlled meta-analysis of birds and bats. *Comp. Biochem. Physiol. A Mol. Integr. Physiol.* 235:193–201.

Nespolo RF, González-Lagos C, Solano-Iguaran JJ, Elfwing M, Garitano-Zavala A, Mañosa S, Alonso JC, Altimiras J. 2018. Aerobic power and flight capacity in birds: a phylogenetic test of the heart-size hypothesis. *Journal of Experimental Biology*.

Nudds RL, Bryant DM. 2000. The energetic cost of short flights in birds. *Journal of Experimental Biology* 203:1561–1572.

Rayner JM V. 1988. Form and Function in Avian Flight. In: Current Ornithology. Boston, MA: Springer US. p. 1–66.

Vincze O, Vágási CI, Pap PL, Palmer C, Møller AP. 2018. Wing morphology, flight type and migration distance predict accumulated fuel load in birds. *Journal of Experimental Biology*.
