# Supplementary material for: Flight style and metabolism shape the tempo of genome evolution in birds
Source: PLoS Biol. 2026 Jul 14;24(7):e3003884. doi: 10.1371/journal.pbio.3003884 (PMC13367715; doi:10.1371/journal.pbio.3003884)
Supplement: Peer review history

# Editor report: Decision Letter 0

**DOI:** 10.1371/journal.pbio.3003884.r001

**Authors:** Roland Roberts

19 Dec 2025

Dear Dr Ji,

Thank you for submitting your manuscript entitled "Flight and metabolism shape the tempo of genome evolution in birds" for consideration as a Research Article by PLOS Biology.

Your manuscript has now been evaluated by the PLOS Biology editorial staff, and I'm writing to let you know that we would like to send your submission out for external peer review.

IMPORTANT: We think that your paper would be best considered as a Short Report. No re-formatting is required, but please select"Short Reports" as the article type when you upload your additional metadata (see next paragraph).

However, before we can send your manuscript to reviewers, we need you to complete your submission by providing the metadata that is required for full assessment. To this end, please login to Editorial Manager where you will find the paper in the 'Submissions Needing Revisions' folder on your homepage. Please click 'Revise Submission' from the Action Links and complete all additional questions in the submission questionnaire.

Once your full submission is complete, your paper will undergo a series of checks in preparation for peer review. After your manuscript has passed the checks it will be sent out for review. To provide the metadata for your submission, please Login to Editorial Manager (https://www.editorialmanager.com/pbiology) within two working days, i.e. by Dec 21 2025 11:59PM.

If your manuscript has been previously peer-reviewed at another journal, PLOS Biology is willing to work with those reviews in order to avoid re-starting the process. Submission of the previous reviews is entirely optional and our ability to use them effectively will depend on the willingness of the previous journal to confirm the content of the reports and share the reviewer identities. Please note that we reserve the right to invite additional reviewers if we consider that additional/independent reviewers are needed, although we aim to avoid this as far as possible. In our experience, working with previous reviews does save time.

If you would like us to consider previous reviewer reports, please edit your cover letter to let us know and include the name of the journal where the work was previously considered and the manuscript ID it was given. In addition, please upload a response to the reviews as a 'Prior Peer Review' file type, which should include the reports in full and a point-by-point reply detailing how you have or plan to address the reviewers' concerns.

During the process of completing your manuscript submission, you will be invited to opt-in to posting your pre-review manuscript as a bioRxiv preprint. Visit http://journals.plos.org/plosbiology/s/preprints for full details. If you consent to posting your current manuscript as a preprint, please upload a single Preprint PDF.

Feel free to email us at plosbiology@plos.org if you have any queries relating to your submission.

Kind regards,

Roli Roberts

Roland Roberts, PhD

Senior Editor

PLOS Biology

rroberts@plos.org

# Peer review: Decision Letter 1

**DOI:** 10.1371/journal.pbio.3003884.r002

**Authors:** Roland Roberts, Roland Roberts

11 Mar 2026

Dear Dr Ji,

Thank you for your patience while your manuscript "Flight and metabolism shape the tempo of genome evolution in birds" was peer-reviewed at PLOS Biology. Please note that I am currently handling your manuscript since my colleague Roland Roberts is out of the office this week. I am very sorry for the delays that you have experienced during the peer review process. Your manuscript has now been evaluated by the PLOS Biology editors, an Academic Editor with relevant expertise, and by two independent reviewers.

In light of the reviews, which you will find at the end of this email, we would like to invite you to revise the work to thoroughly address the reviewers' reports.

As you will see, the reviewers agree that your study is interesting but they raise several important concerns that should be addressed in a revised version. Reviewer #1 notes that the analyses do not appear to consider the potential influence of effective population sizes on substitution rates and asks that this is included. He/she also asks that diversifications rates are incorporated into the modelling framework. After discussions with the Academic Editor, we think it would also be beneficial to include additional analyses directly comparing per generation mutation rates. In addition, Reviewer #2 asks that the settings for the AIC test are confirmed and raises concerns with the clarity of the Methods section and the flight style designations.

Given the extent of revision needed, we cannot make a decision about publication until we have seen the revised manuscript and your response to the reviewers' comments. Your revised manuscript is likely to be sent for further evaluation by all or a subset of the reviewers.

In addition to these revisions, you will need to complete some formatting changes, which you will receive in a follow up email. A member of our team will be in touch with a set of requests shortly.

We expect to receive your revised manuscript within 3 months. Please email us (plosbiology@plos.org) if you have any questions or concerns, or would like to request an extension.

At this stage, your manuscript remains formally under active consideration at our journal; please notify us by email if you do not intend to submit a revision so that we may withdraw it.

**IMPORTANT - SUBMITTING YOUR REVISION**

Your revisions should address the specific points made by each reviewer. Please submit the following files along with your revised manuscript:

1. A 'Response to Reviewers' file - this should detail your responses to the editorial requests, present a point-by-point response to all of the reviewers' comments, and indicate the changes made to the manuscript.

*NOTE: In your point-by-point response to the reviewers, please provide the full context of each review. Do not selectively quote paragraphs or sentences to reply to. The entire set of reviewer comments should be present in full and each specific point should be responded to individually, point by point.

You should also cite any additional relevant literature that has been published since the original submission and mention any additional citations in your response.

2. In addition to a clean copy of the manuscript, please also upload a 'track-changes' version of your manuscript that specifies the edits made. This should be uploaded as a "Revised Article with Changes Highlighted" file type.

*Re-submission Checklist*

When you are ready to resubmit your revised manuscript, please refer to this re-submission checklist: https://plos.io/Biology_Checklist

To submit a revised version of your manuscript, please go to https://www.editorialmanager.com/pbiology/ and log in as an Author. Click the link labelled 'Submissions Needing Revision' where you will find your submission record.

Please make sure to read the following important policies and guidelines while preparing your revision:

*Published Peer Review*

Please note while forming your response, if your article is accepted, you may have the opportunity to make the peer review history publicly available. The record will include editor decision letters (with reviews) and your responses to reviewer comments. If eligible, we will contact you to opt in or out. Please see here for more details:

https://blogs.plos.org/plos/2019/05/plos-journals-now-open-for-published-peer-review/

*PLOS Data Policy*

Please note that as a condition of publication PLOS' data policy (http://journals.plos.org/plosbiology/s/data-availability) requires that you make available all data used to draw the conclusions arrived at in your manuscript. If you have not already done so, you must include any data used in your manuscript either in appropriate repositories, within the body of the manuscript, or as supporting information (N.B. this includes any numerical values that were used to generate graphs, histograms etc.). For an example see here: http://www.plosbiology.org/article/info%3Adoi%2F10.1371%2Fjournal.pbio.1001908#s5

*Blot and Gel Data Policy*

We require the original, uncropped and minimally adjusted images supporting all blot and gel results reported in an article's figures or Supporting Information files. We will require these files before a manuscript can be accepted so please prepare them now, if you have not already uploaded them. Please carefully read our guidelines for how to prepare and upload this data: https://journals.plos.org/plosbiology/s/figures#loc-blot-and-gel-reporting-requirements

*Protocols deposition*

To enhance the reproducibility of your results, we recommend that if applicable you deposit your laboratory protocols in protocols.io, where a protocol can be assigned its own identifier (DOI) such that it can be cited independently in the future. Additionally, PLOS ONE offers an option for publishing peer-reviewed Lab Protocol articles, which describe protocols hosted on protocols.io. Read more information on sharing protocols at https://plos.org/protocols?utm_medium=editorial-email&utm_source=authorletters&utm_campaign=protocols

Thank you again for your submission to our journal. We hope that our editorial process has been constructive thus far, and we welcome your feedback at any time. Please don't hesitate to contact us if you have any questions or comments.

Best regards,

Richard

Richard Hodge, PhD

Senior Editor, PLOS Biology

rhodge@plos.org

On behalf of:

Roland Roberts, PhD

Senior Editor, PLOS Biology

rroberts@plos.org

------------------------------------

REVIEWS:

Reviewer #1: In this study the authors consider how various ecological and life history traits effect rates of evolution in birds. The results of the study are intriguing, and it is overall an excellent study. I only have a few suggestions.

1.First off, I would strongly suggest including a table that give readers an idea of the relative influence of life history traits vs the other traits that the authors consider, since the current paradigm is that substitution rates are determined largely by generation time. For example, my interpretation of Fig. 2 A-C would be that most of the relationship between body size and substitution rates is driven by larger species having longer generation time.

2.Another thing that surprised me was that the authors did not seemingly consider the potential influence of effective population size on rates of substitution. For example a fairly recent high profile study (Bergeron et al. 2023) showed evidence that this factor influences rates of evolution across all vertebrates. Stiller et al (2024), which the authors cite and includes at least one shared author that I spotted (and I assume I am missing a few more!) also calculated effective population size across birds, so it seems that it would be relatively straightforward to include it. At the very least, Bergeron should be cited and the authors should make it clear why they didn't include it. On the other hand, if that is one of the "life history traits" that is included in the model only shown in the supplement, my first comment goes double!

3.Another factor that one might expect to be related to rates of evolution is diversification rate. In fact, there is a major debate about whether diversification rates drive variation in rates of morphological trait evolution (e.g., Adams et al. 2009 vs Rabosky et al. 2013). I think there would be some reader interest in knowing what is going on with diversification rates and rates of molecular evolution.

If the authors wanted to include this, tip level metrics of diversification rate are easily to calculate, and would be straightforward to incorporate into their species level modelling framework. See, for example, the method used in the classic Jetz et al. (2012) study, which also has a tree they could use to calculate accurate rates (since a tree that doesn't include all birds might give incorrect rates).

4.Another small quibble is that the authors did not make it clear that they adjusted their PGLS analysis for the amount of phylogenetic signal observed in uncorrected model residuals. Since the authors used nlme for this analysis, I assumed that they used the corBrownian function to do just that. However, readers not intimately familiar with that R package might not realize it.

A brief note "using the corBrownian function to adjust each model for the amount of phylogenetic signal observed in residuals" around line 210 is all that is needed.

5.Finally, in the discussion the authors may want to reference the metabolic theory of ecology (Brown 2004, Sibley et al. 2012), since their results broadly agree with it. Even though the authors did not attempt to specifically evaluate whether quarter power scaling was present, this is one of the clearest demonstrations that I am aware of that metabolic rates influence sequence substitution rates even after accounting for life history variation. I think that crowd will be excited by the result.

All that said, in case it is not clear at this point, let me say again that this is an excellent analysis. I have no issues with the conclusions that the authors have drawn, or the methods that they chose to use.

Cited:

Adams, D. C., Berns, C. M., Kozak, K. H., & Wiens, J. J. (2009). Are rates of species diversification correlated with rates of morphological evolution?. Proceedings of the Royal Society B: Biological Sciences, 276(1668), 2729-2738.

Bergeron, L. A., Besenbacher, S., Zheng, J., Li, P., Bertelsen, M. F., Quintard, B., ... & Zhang, G. (2023). Evolution of the germline mutation rate across vertebrates. Nature, 615(7951), 285-291.

Brown, J. H., Gillooly, J. F., Allen, A. P., Savage, V. M., & West, G. B. (2004). Toward a metabolic theory of ecology. Ecology, 85(7), 1771-1789.

Jetz, W., Thomas, G. H., Joy, J. B., Hartmann, K., & Mooers, A. O. (2012). The global diversity of birds in space and time. Nature, 491(7424), 444-448.

Rabosky, D. L., Santini, F., Eastman, J., Smith, S. A., Sidlauskas, B., Chang, J., & Alfaro, M. E. (2013). Rates of speciation and morphological evolution are correlated across the largest vertebrate radiation. Nature communications, 4(1), 1958.

Sibly, R. M., Brown, J. H., & Kodric-Brown, A. (Eds.). (2012). Metabolic ecology: a scaling approach. John Wiley & Sons.

Stiller, J., Feng, S., Chowdhury, A. A., Rivas-González, I., Duchêne, D. A., Fang, Q., ... & Zhang, G. (2024). Complexity of avian evolution revealed by family-level genomes. Nature, 629(8013), 851-860.

Reviewer #2: As data on molecular rates becomes more available, there is an increasing field that examines the predictors of these rates using comparative data. Several papers have considered flight as a potential predictor of evolutionary rates, due to its potential connections with metabolism. This paper adds to that literature. The results are potentially interesting but I see several areas where improvement or further examination is needed:

First, the study uses AIC model comparison with PGLS models, using the gls function. It is important when doing this that the maximum likelihood estimator for the variance is set to "ML" and not the default "REML" for this function, which results in models that cannot be compared. The paper does not specify if this was done, and I could not access the zenodo link to check the code myself. Please confirm and clarify.

Second, the Materials and Methods section is written in a very confusing way. I think that what happened is that the authors wrote the paper for a journal such as PNAS in which the Results and Discussion come before the Methods, but then they moved the Methods before the results for this submission without editing it carefully to include the necessary information in Methods before the Results section. Consequently, there were several areas where they did not include important information in the Methods section that was later presented in Results. This needs to be reorganized for readability.

Third, I found the flight style designations confusing. I am surprised that so many passerines were classified as undulating rather than continuous flight -- this does not seem right to me. I also was not sure what flapping and gliding meant. I could not easily find a list of species and their category, so this was hard to evaluate. Given that there differences in the evolutionary rates for undulating but not continuous flight, this needs critical examination, and probably fewer categories are better. Additionally, I found that the addition of the Aerial Lifestyle Index did not add anything useful to the paper, and probably just added an extra layer of complexity that made the results harder to interpret. I would recommend removing this.

Fourth, the paper rests on an estimate of evolutionary rates that is a root to tip distance based on an ancestral state reconstruction. But, of course, the ancestral state estimation could be affected by heterogeneity in evolutionary rates. How this kind of circularity could affect the estimation of rates is not clear to me, but I think it deserves mention and discussion.

Finally, I thought the paper did a rather poor job of explaining WHY differences in flight style might result in differences in evolutionary rate. Why should we see higher rates in undulating flight species but not continuously flapping? (e.g., Figure 2). If you are going to make these categorizations, then there should be an hypothesis or predictions for how and why the variation should affect the outcome variable, and I don't see that here. I can imagine metabolic differences between, say, flapping and soaring species, but the finer-scale designations among flapping species are much more tenuously connected to metabolism, if at all. This also relates to my third comment above. As such, the results are difficult to connect to biology.

# Author comment: Author response to Decision Letter 2

**DOI:** 10.1371/journal.pbio.3003884.r003

13 May 2026

Attachment Submitted filename: Comments_PB_final.pdf

# Peer review: Decision Letter 2

**DOI:** 10.1371/journal.pbio.3003884.r004

**Authors:** Roland Roberts, Roland Roberts, Roland Roberts

17 Jun 2026

Dear Dr Ji,

Thank you for the submission of your revised Short Report "Flight style and metabolism shape the tempo of genome evolution in birds" for publication in PLOS Biology. On behalf of my colleagues and the Academic Editor, Laurence Hurst, I'm pleased to say that we can in principle accept your manuscript for publication, provided you address any remaining formatting and reporting issues. These will be detailed in an email you should receive within 2-3 business days from our colleagues in the journal operations team; no action is required from you until then. Please note that we will not be able to formally accept your manuscript and schedule it for publication until you have completed any requested changes.

IMPORTANT:

a) We obtained new comments from reviewer #1, who said "I thought that the first version of the manuscript was a strong contribution. The authors have incorporated all of the revisions I suggested, and in the process both clarified the manuscript and connected it more clearly to previous work. I have no further suggested revisions." Reviewer #2 was unable to re-review. I discussed these comments with the Academic Editor, who was happy to recommend publication.

b) I have taken the liberty of making a very minor change to your Title (the insertion of the word "style") for clarity.

c) In order to expedite the processing of the manuscript, I have also included the following data citation in all relevant Figure legends: "The data and code required to generate this Figure can be found in https://doi.org/10.5281/zenodo.20093624"

Please take a minute to log into Editorial Manager at http://www.editorialmanager.com/pbiology/, click the "Update My Information" link at the top of the page, and update your user information to ensure an efficient production process.

PRESS: We frequently collaborate with press offices. If your institution or institutions have a press office, please notify them about your upcoming paper at this point, to enable them to help maximise its impact. If the press office is planning to promote your findings, we would be grateful if they could coordinate with biologypress@plos.org. If you have previously opted in to the early version process, we ask that you notify us immediately of any press plans so that we may opt out on your behalf.

We also ask that you take this opportunity to read our Embargo Policy regarding the discussion, promotion and media coverage of work that is yet to be published by PLOS. As your manuscript is not yet published, it is bound by the conditions of our Embargo Policy. Please be aware that this policy is in place both to ensure that any press coverage of your article is fully substantiated and to provide a direct link between such coverage and the published work. For full details of our Embargo Policy, please visit http://www.plos.org/about/media-inquiries/embargo-policy/.

Thank you again for choosing PLOS Biology for publication and supporting Open Access publishing. We look forward to publishing your study.

Sincerely,

Roli Roberts

Roland G Roberts, PhD, PhD

Senior Editor

PLOS Biology

rroberts@plos.org
